# Evidence Synthesis for Complex Interventions Using Meta-Regression Models

**DOI:** 10.1093/aje/kwad184

**Published:** 2023-09-08

**Authors:** Kristin J Konnyu, Jeremy M Grimshaw, Thomas A Trikalinos, Noah M Ivers, David Moher, Issa J Dahabreh

**Keywords:** complex interventions, hierarchical models, meta-analysis, meta-regression, multicomponent interventions

## Abstract

A goal of evidence synthesis for trials of complex interventions is to inform the design or implementation of novel versions of complex interventions by predicting expected outcomes with each intervention version. Conventional aggregate data meta-analyses of studies comparing complex interventions have limited ability to provide such information. We argue that evidence synthesis for trials of complex interventions should forgo aspirations of estimating causal effects and instead model the response surface of study results to 1) summarize the available evidence and 2) predict the average outcomes of future studies or in new settings. We illustrate this modeling approach using data from a systematic review of diabetes quality improvement (QI) interventions involving at least 1 of 12 QI strategy components. We specify a series of meta-regression models to assess the association of specific components with the posttreatment outcome mean and compare the results to conventional meta-analysis approaches. Compared with conventional approaches, modeling the response surface of study results can better reflect the associations between intervention components and study characteristics with the posttreatment outcome mean. Modeling study results using a response surface approach offers a useful and feasible goal for evidence synthesis of complex interventions that rely on aggregate data.

## Abbreviations

CrI: credible interval
HbA1c: hemoglobin A1c
ICC: intraclass correlation coefficient
QI: quality improvement

Interventions designed to change health-care practice and health policy are often “complex” in that they involve multiple components delivered to multiple levels of health care (e.g., patients, providers, clinics) and across multiple settings (1, 2). Although researchers continue to refine the definition of a complex intervention (1–5), most definitions require 3 features: 1) complex interventions involve combinations of several more-elemental intervention “components”; 2) there can be intricate interactions between the components and/or any ancillary cointerventions; and 3) the components may have effects that vary with study-specific characteristics, such as health-care settings and patient populations—that is, the components may exhibit different treatment effect heterogeneity patterns (6, 7).

The above way of thinking about complex interventions is intuitive and not particularly restrictive. It can describe interventions considered for a wide range of policy, health-care delivery, and operations management problems, from what behavioral measures to roll out in the beginning of the coronavirus disease 2019 epidemic to managing patients with diabetes (8), developing decision aids for patients facing difficult decisions (9), or proposing combination chemotherapy regimens to be evaluated in future trials (10). It also suggests an obvious organizing scheme for describing how a complex intervention works and theorizing about which modifications might be improvements. Behavioral-intervention theorists and implementation scientists often employ similar modular approaches to designing their interventions (11, 12).

A naturally arising need is to predict average outcomes with different versions of a complex intervention in a target setting. Prediction of average outcomes sets a lower bar than estimation of causal effects. Except for narrow questions, results of evidence synthesis of aggregate data do not have a clear causal interpretation. Causally interpretable meta-analyses involve estimating the effects of well-defined interventions in a well-defined target population—a challenging task that requires strong structural assumptions and rich individual participant data (13, 14).

Our emphasis on prediction of posttreatment average outcomes with complex interventions differs from the stated goals of other methodological (15, 16) and applied (17–23) works that aim to estimate causal effects for each component of a complex intervention by modeling the between-arm differences in each trial. Other authors have proposed the use of multivariable meta-regression models as an alternative synthesis approach for complex interventions (24, 25), reframing the goal of synthesis away from estimation of any single causal effect to the estimation of a *response surface* (25) conditional on the individual intervention components and on population and setting characteristics of interest. In this paper, we adopt the latter view: We argue that in most cases, particularly when using aggregate data from very diverse studies, the goal of evidence synthesis should be not to estimate a single causal effect but to estimate a function of the different components and, potentially, their interactions with population characteristics and contextual factors, that can be used to predict the average outcomes of a complex intervention of interest in a future setting or study.

We illustrate an application of the above in a systematic review and meta-analysis of complex interventions for quality improvement (QI) of diabetes management. We first introduce the application and discuss limitations of conventional meta-analysis. We then develop and apply response-surface meta-regression models and discuss estimation, prediction, and ranking. We examine alternative model specifications that allow interactions among intervention components and with study-level covariates. We also discuss handling of discrete outcomes and missing data, including missing estimates of the intraclass correlation coefficient (ICC), to adjust variance data in cluster-randomized trials. Last, we discuss the strengths and limitations of our approach.

## MOTIVATING EXAMPLE: QI INTERVENTIONS TO IMPROVE DIABETES CARE

The International Diabetes Federation estimates that 415 million adults were living with diabetes in 2015 and predicts that the prevalence will exceed 640 million by 2040 (26). People living with diabetes are at increased risk for serious complications, such as cardiovascular events and blindness. Despite evidence that clinical interventions such as monitoring glycemic control, monitoring microvascular complications, and managing vascular risk factors improve patient outcomes and reduce costs (27, 28), many patients with diabetes do not receive evidence-based care and have suboptimal control of risk factors (29, 30). Diabetes QI interventions seek to address these evidence-to-practice gaps by targeting system, provider, or patient factors influencing diabetes care (8). These QI interventions fit our definition of complex interventions—their different versions are combinations of a subset of 12 more-elemental components (8).

As an example, we use data from a systematic review by Tricco et al. (8) that examined evidence from 142 randomized trials of the effects of QI interventions (comprised of component QI strategies) on a range of procedural (e.g., foot screening) and intermediate patient (e.g., glycemic control) outcomes. The review codes QI interventions using a taxonomy of 12 component strategies, adapted from Cochrane’s Effective Practice and Organization of Care 2002 taxonomy (Table 1) (8, 31, 32). Most included trials evaluated intervention versions with a median of 3 QI components (range, 1–8). For the main outcome, levels of hemoglobin A1c (HbA1c; a measure of glycemic control), the authors found that in 120 trials that compared using a QI intervention with not using one, the average mean reduction in HbA1c levels was 0.37% (95% confidence interval: 0.28, 0.45) in random-effects meta-analysis, but with evidence of substantial heterogeneity (*I*^2^ statistic = 73%). Analyses assessing the efficacy of QI interventions containing a specific component of interest (e.g., case management), as compared with QI interventions not containing that component (e.g., no case management), found improvements associated with most QI components but could not disentangle the effects of co-occurring components. Finally, the meta-analysis did not assess nonadditivity in the relationship between intervention components and the posttreatment outcome mean or examine modification of the association between each intervention component and the posttreatment outcome mean (also referred to as “moderation”) by study-level covariates. Thus, despite the ostensibly large number of studies, the authors of the review could not explain the observed heterogeneity or predict the outcomes of novel combinations of components in a new population or setting.

**Table 1:** Taxonomy of Quality Improvement Strategies Adapted From Cochrane’s Effective Practice and Organization of Care 2002 Taxonomy That Were Used to Code Quality Improvement Interventions

**QI Strategy**	**Definition**
Audit and feedback	Summary of clinical performance of health care delivered by an individual clinician or clinic over a specified period, transmitted back to the clinician
Case management	Any system for coordinating diagnosis, treatment, or routine management of patients by a person or multidisciplinary team in collaboration with, or supplementary to, the primary-care clinician
Team changes	Changes to the structure or organization of the primary health-care team, including adding a team member or shared care, use of multidisciplinary teams, or expansion or revision of professional roles
Electronic patient registry	General electronic medical record system or electronic tracking system for patients with the condition
Clinician education	Interventions designed to promote increased understanding of principles guiding clinical care or awareness of specific recommendations for a target disorder or population of patients
Clinician reminders	Paper-based or electronic systems intended to prompt a health professional to recall patient-specific information
Facilitated relay of clinical information	Clinical information collected from patients and transmitted to clinicians by means other than the existing medical record
Patient education	Interventions designed to promote greater understanding of a target disorder or to teach specific prevention or treatment strategies, or specific in-person education
Promotion of self- management	Provision of equipment or access to resources to promote self-management
Patient reminders	Any effort to remind patients about upcoming appointments or important aspects of self-care
Continuous quality improvement	Interventions explicitly identified as involving the techniques of continuous QI, total quality management, or plan-do-study-act, or any iterative process for assessing quality problems, developing solutions to those problems, testing their effects, and then reassessing the need for further action
Financial incentives	Interventions with positive or negative financial incentives directed at providers or patients or systemwide changes in reimbursement

Abbreviation: QI, quality improvement.

## PROBLEMS WITH CONVENTIONAL PAIRWISE META-ANALYSIS

**Figure 1:**
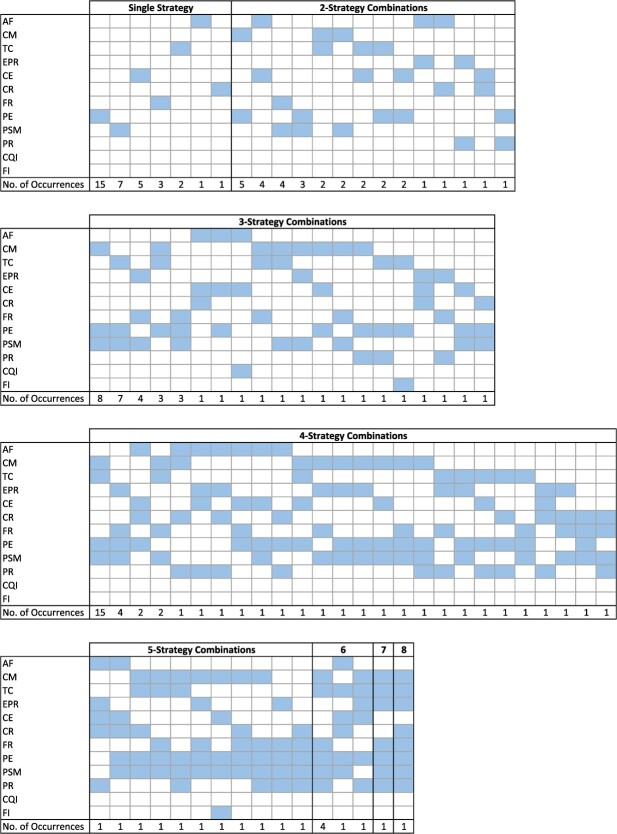
Frequency of evaluations of quality improvement (QI) interventions comprised of component QI strategies. AF, audit and feedback; CE, clinician education; CM, case management; CR, clinician reminder; EPR, electronic patient registry; FR, facilitated relay; PE, patient education; PR, patient reminders; PSM, promotion of self-management; TC, team changes.

 One important problem in meta-analyses of complex interventions is that the empirical evidence is often sparse. The number of possible versions of complex interventions grows exponentially with the number of components. Assuming we can meaningfully combine *m* components without constraints, we can create ${2}^m-1$ nonempty versions of a complex intervention. This number is 4,095 in our QI example—an order of magnitude more than the total number of available studies. Moreover, only a handful of the possible versions are observed in the empirical data. For the HbA1c outcome, only 83 unique interventions were observed (about 2% of the possible ones), and 59 of these were assessed in only 1 trial (Figure 1**)**.

Comparing any version of a QI intervention with doing nothing in a pairwise meta-analysis may have some descriptive value but has little practical usefulness (8). Such a meta-analysis involves an ill-defined comparison: The experimental arm involves doing *something* (from a rather mixed bag of somethings) versus nothing—and the target population is not precisely defined. This question reduces a complicated problem to a simple one, at the cost of obtaining uninformative results: A difference between doing something and doing nothing tells us nothing about which version of the complex intervention a policy-maker should choose. Conversely, finding no difference on average does not imply that all versions of the complex intervention are ineffective.

An additional problem with commonly used meta-analytical approaches is that they do not use all available information. Some trials of complex interventions have 3 or more arms (3, 33), of which 2 are typically selected for a meta-analysis. For example, when comparing any active intervention with nothing, analysts often use only 2 arms from each study (i.e., complex interventions with the greatest number of components vs. the least number of components). This further complicates the interpretability of the findings, because the most intensive complex intervention in one study can be the least intensive intervention in another. Additional problems involve missing estimates of the sampling variance or missing estimates of the ICC in cluster-randomized trials that are needed to adjust unadjusted estimates of the sampling variance.

To obtain useful information about any version of the complex intervention, we must *extrapolate*, through statistical modeling, from the observed versions to the unobserved ones, using *all available information*. From the point of view of learning a response surface described above (i.e., that maps combinations of components and settings to average outcomes), using all available information amounts to *modeling outcomes of all arms in all studies*, as described below.

## SPECIFICATION OF THE BASIC RESPONSE SURFACE META-REGRESSION MODEL AND OF PRIOR DISTRIBUTIONS AND INFERENCE

### Specification of the response surface model

We model the associations of intervention components and study- or arm-level modifiers with the posttreatment outcome means using a random-effects regression with heteroskedastic errors (a hierarchical meta-regression model) (24, 25, 34, 35).

Let ${Y}_{ij}$ be the posttreatment mean in the *j*th arm of the *i*th study, distributed as

(1) \begin{equation*} {Y}_{ij}\sim N\left({\mathrm{\mu}}_{ij},{\mathrm{\theta}}_{ij}^2\right),i=1,\dots, n;j=1,\dots{n}_i, \end{equation*}

where ${\mathrm{\mu}}_{ij}$ is the arm-specific true mean and ${\mathrm{\theta}}_{ij}^2$ is the conditional (sampling) variance. Given the large number of components and potential interactions between them, we will usually have to assume a parsimonious model for ${\mathrm{\mu}}_{ij}$. We begin by considering a linear additive model in terms of the *m* components:

(2) \begin{equation*} {\mathrm{\mu}}_{ij}={\mathrm{\beta}}_{0i}+\sum \limits_{k=1}^m{\mathrm{\beta}}_{ki}{X}_{ij k}, \end{equation*}

where ${X}_{ijk}$ denotes the value of the *k*th component (coded as 0 if absent or 1 if present) in the *j*th arm of the *i*th study. Henceforth, we refer to the coefficients ${\mathrm{\beta}}_{ki}$ as “mean differences” because they express the difference in the posttreatment mean when component ${X}_{ijk}$ is present but make no causality claims. The intercept, ${\mathrm{\beta}}_{0i}$, represents the posttreatment mean in the absence of intervention (36). ${\mathrm{\beta}}_{0i}$ and ${\mathrm{\beta}}_{ki}$ are treated as study-specific nuisance parameters in the estimation. We assume that they are realizations of underlying random variables, each with a normal distribution

(3) \begin{equation*} {\mathrm{\beta}}_{ki}\sim N\left({\mathrm{\beta}}_k,{\mathrm{\tau}}_k^2\right),k=1,\dots, m \end{equation*}

whose mean ${\mathrm{\beta}}_k$ and variance ${\mathrm{\tau}}_k^2$ will be estimated from the model. Study intercepts, ${\mathrm{\beta}}_{0i}$, are also assumed to be random variables with a normal distribution with mean ${\mathrm{\beta}}_0$ and variance ${\mathrm{\tau}}_0^2$,

(4) \begin{equation*} {\mathrm{\beta}}_{0i}\sim N\left({\mathrm{\beta}}_0,{\mathrm{\tau}}_0^2\right). \end{equation*}

Equation 3 assumes exchangeable arm-specific parameters. We believe that this is justified in meta-analyses of trials of complex interventions where studies commonly evaluate active arms, the distinction between intervention and control arms is often unclear, and it is common for some intervention components to be assessed in each arm. In addition, for simplicity, equation 3 uses independent normal distributions. We believe that this is justified for our application because intervention components often operate at different levels (e.g., health system, provider, patient), and it is likely that their distribution would not involve strong correlation. If such correlations were in evidence in the data, they would be revealed in the estimated joint distribution of the β’s. Alternatively, note that equation 3 is equivalent to an *m*-variate normal distribution with a diagonal covariance matrix, and, if desired, one could assume correlated β’s with various structures for the covariance matrix (e.g., unstructured, compound-symmetrical, or other, depending on topic-specific information).

### Specification of prior distributions

Some features of our approach, such as incorporating external information and handling of missing data (see “Application to the Diabetes QI Example”), are most naturally achieved in the Bayesian framework (37, 38). Furthermore, Bayesian hierarchical modeling more fully reflects parameter uncertainty and is appealing for evidence synthesis because the study similarity judgments that systematic reviewers make are conceptually related to Bayesian exchangeability assumptions (39). For these reasons, we opted to use a Bayesian approach to estimate model parameters. Specifically, we used the minimally informative normal distributions for ${\mathrm{\beta}}_0$ and ${\mathrm{\beta}}_k$ and uniform distributions for ${\mathrm{\tau}}_k$ and ${\mathrm{\tau}}_0$ (see the Discussion section). Systematic reviewers can often rely on other meta-analyses or expert opinion to specify prior distributions to improve estimation with sparse data (37). Use of informative prior distributions is particularly useful for the heterogeneity parameters, ${\mathrm{\tau}}_k^2$, which are often poorly estimated (40, 41).

### Inference

We can use the model in “Specification of the response surface model” to 1) estimate the posterior distribution of the mean difference for each intervention component and make inferences from that posterior distribution; 2) rank the components by the magnitude of the mean differences—that is, estimate the probability that the mean difference for a component has the greatest mean difference, the second greatest, and so on among the components included in the model (42); and 3) predict the posttreatment mean in future studies, possibly using combinations of components that have not been previously assessed in trials (43). For example, predictive inference can be obtained by examining the posterior predictive distribution for the posttreatment mean for any combination of components

(5) \begin{equation*} {\mathrm{\mu}}_{\mathrm{new}}={\mathrm{\beta}}_{0,\mathrm{new}}+\sum \limits_{k=1}^m\ {\mathrm{\beta}}_{k,\mathrm{new}}{X}_{k,\mathrm{new}} \end{equation*}

and

(6) \begin{equation*} {\mathrm{\beta}}_{k,\mathrm{new}}\sim N\left({\mathrm{\beta}}_k,{\mathrm{\tau}}_{\mathrm{\beta} k}^2\right),k=0,\dots, m, \end{equation*}

where ${X}_{k,\mathrm{new}}$ denotes the *k*th component in the new study. The posterior predictive distribution of ${\mathrm{\mu}}_{\mathrm{new}}$ can be used when designing a new study, because planning decisions can be based on the posterior predictive distribution for a particular study,

(7) \begin{equation*} {Y}_{\mathrm{new}}\sim N\left({\mathrm{\mu}}_{\mathrm{new}},{\mathrm{\theta}}_{\mathrm{new}}^2\right), \end{equation*}

where ${\mathrm{\theta}}_{\mathrm{new}}^2$ denotes the sampling variance of the new study, which depends on the planned sample size.

### Model extensions

Web Appendix 1 (available at https://doi.org/10.1093/aje/kwad184) extends the model to include pairwise product terms among components and between components and study-level covariates, handle discrete outcomes and missing data, and impute missing estimates of the ICC to adjust variance data in cluster-randomized trials.

## APPLICATION TO THE DIABETES QI EXAMPLE

We implemented a series of response surface models to describe the associations of each QI component with average outcomes and compared them to estimates from the conventional meta-analysis model. We used data from 114 trials (241 arms, 48,969 patients) that reported mean HbA1c levels at baseline and postintervention obtained from the 2012 version of the review (8), which is in the process of being updated (44). While updating the original review, we revised some data extraction algorithms, which resulted in small changes to the data (44). Because of these changes, our results are similar but not identical to those reported in 2012. We imputed missing information on standard errors of posttreatment arm means in individually randomized trials and missing intracluster correlation coefficients in cluster- randomized trials as per Web Appendix 2. To facilitate modeling, and with content-expert input, we combined QI components that were observed infrequently (present in less than 10% of arms) into an “other” category. This category included the QI components for clinician reminders (9.5%), audit and feedback (8.7% of arms), financial incentives (0.8%), and continuous QI (0.4%).

The conventional meta-analysis model was fitted in R (overall and subgroup analyses for each QI component) using the “meta” package in R (45); estimates are reported as mean changes and 95% confidence intervals. Hierarchical models were fitted using Markov chain Monte Carlo methods with the software JAGS (46) called from R, with 100,000 iterations for burn-in and 100,000 iterations to obtain the posterior distribution of parameters of interest. We assigned normal prior distributions for the coefficients ${\mathrm{\beta}}_k\sim N\left(0,4\right)$ and the baseline intercept ${\mathrm{\beta}}_0\sim N\left(8,100\right)$ and uniform prior distributions for the between-studies standard deviations ${\mathrm{\tau}}_0,{\mathrm{\tau}}_k\sim U\left(0,2\right)$. Estimates are reported as median changes and 95% credible intervals (CrIs), representing the mean difference associated with the presence of the component relative to the component’s not being present. We used the Brooks-Gelman-Rubin diagnostic to assess parameter convergence (47, 48).

### Comparison of meta-regression and conventional synthesis models

We compared parameter estimates from 3 analyses:

- *Analysis I* imitated commonly used analyses in reviews of complex interventions in using only 2 arms from each trial, selecting the most and least intensive ones in multiarm trials (228 arms; 44,375 individuals). It comprised 1 random-effects meta-regression per QI component, where the sole predictor was the presence or absence of the QI component in the experimental arm. The model for the *k*th component did not adjust for the remaining components.
- *Analysis II* used the same 2 arms from each trial as analysis I in a single random-effects meta-regression that fitted the response surface model in equations 1–4.
- *Analysis III* used all arms in all trials in a meta-regression according to the model in equations 1–4 (241 arms; 48,969 individuals).

In analysis I, we imputed missing standard deviations and ICCs with fixed values (2.22 for standard deviations, the 99th percentile in observed data; and 0.027 for ICCs, obtained from a single study), as was done in the original review (49). We used ICCs to correct standard errors from cluster-randomized trials in which results were not appropriately adjusted for the clustering effect (49). Sensitivity analyses using a less conservative standard deviation (median, 1.34) and the higher ICC used in the 2012 review (8) (ICC = 0.07) did not change the overall mean and precision of the random-effects meta-analysis appreciably. Missing data in analyses II and III were imputed in the hierarchical meta-regression model as described in Web Appendices 1 and 2. Web Appendix 3 (including Web Tables 1 and 2) describes the different missing data patterns observed in the data set. Software code for all analyses is presented in Web Appendices 4 and 5 and on the authors’ GitHub page (https://github.com/kkonnyu/evsynthmetaregression).

Table 2 summarizes the results. Compared with analysis I, the estimated coefficients for each component were smaller in analysis II and had a wider variation in magnitude. If the models in analyses II and III are approximately correctly specified, the smaller (and more varied) magnitude of the estimates from analyses II and III as compared with the estimates from analysis I may indicate better isolation of the expected posttreatment mean reduction associated with unique QI components, *accounting for co-occurring components*. In other words, analysis I, and thus conventional meta-analysis, would overestimate the associations of a single QI component with differences in outcomes. Point estimates of parameters from analysis II, which used only the most and least intensive arms from each trial, were similar to those of analysis III, which used all arms in all trials. However, estimates were more precise in analysis III.

**Table 2:** Summary of Results Comparing Analysis of Diabetes Quality Improvement Components Using Conventional and Response Surface Meta-Regression Models

	**Analysis and Model**					
	**Analysis I Conventional MA** ^**a**^		**Analysis II Meta-Regression** ^**a**^ ^**,**^ ^**b**^		**Analysis III Meta-Regression** ^**b**^ ^**,**^ ^**c**^	
**QI Strategy**	**MD, % HbA1c**	**95% CI**	**MD, % HbA1c**	**95% CrI**	**MD, % HbA1c**	**95% CrI**
CM	−0.42	−0.55, −0.29	0.03	−0.13, 0.18	0.03	−0.12, 0.17
TC	−0.53	−0.69, −0.37	−0.37	−0.55, −0.19	−0.36	−0.54, −0.18
EPR	−0.37	−0.53, −0.22	−0.16	−0.40, 0.08	−0.15	−0.38, 0.07
CE	−0.23	−0.37, −0.09	−0.18	−0.47, 0.09	−0.17	−0.44, 0.08
FR	−0.40	−0.54, −0.26	−0.23	−0.42, −0.03	−0.24	−0.43, −0.06
PE	−0.44	−0.56, −0.32	−0.05	−0.24, 0.15	−0.10	−0.28, 0.08
PSM	−0.41	−0.52, −0.30	−0.21	−0.41, −0.01	−0.17	−0.37, 0.01
PR	−0.33	−0.53, −0.14	0.03	−0.21, 0.28	−0.00	−0.23, 0.22
Other^d^	−0.19	−0.31, −0.06	−0.01	−0.25, 0.21	0.02	−0.20, 0.18

Abbreviations: CE, clinician education; CI, confidence interval; CM, case management; CrI, credible interval; EPR, electronic patient registry; FR, facilitated relay; HbA1c, hemoglobin A1c; MA, meta-analysis; MD, mean difference; PE, patient education; PR, patient reminders; PSM, promotion of self-management; QI, quality improvement; TC, team changes.

^a^ Analyses used only the most intensive arms of multiple-arm trials versus the least intensive arms.

^b^ The following priors were used in the Bayesian analyses to estimate parameters in analyses I and II: ${\mathrm{\beta}}_0\sim N\left(\mathrm{8,100}\right)$; ${\tilde{\mathrm{\tau}}}_0=U\left(0,2\right)$; ${\mathrm{\beta}}_k\sim N\left(0,4\right)$; and ${\mathrm{\tau}}_{\beta_k}=U\left(0,2\right)$.

^c^ Analyses used all arms from all trials.

^d^ “Other” represents a combined category for infrequently evaluated components, including audit and feedback, clinician reminders, continuous quality improvement, and financial incentives.

### Ranking of coefficients for intervention components

**Figure 2:**
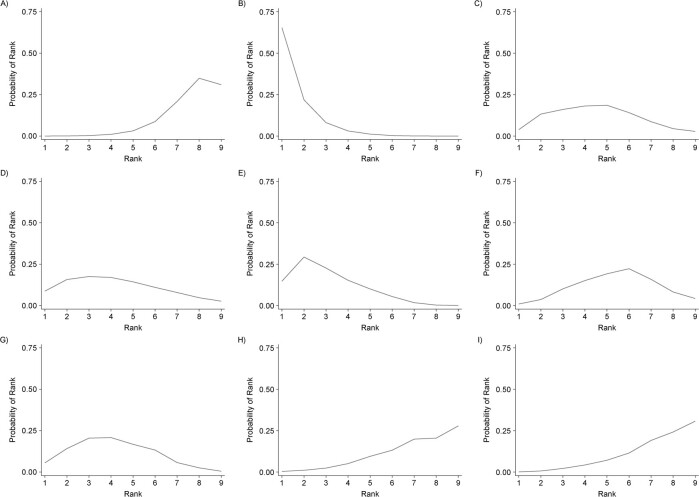
Ranking of quality improvement strategy components. A) Case management; B) team changes; C) electronic patient registry; D) clinician education; E) facilitated relay; F) patient education; G) promotion of self-management; H) patient reminders; I) other. “Other” represents a combined category for infrequently evaluated components, including audit and feedback, clinician reminders, continuous quality improvement, and financial incentives.

An example rankogram that can be produced from estimates of a meta-regression model is presented in Figure 2. Using the output of analysis III above, the rankogram indicates the probability of each component’s being the best, second best, and so on among the modeled components with respect to postintervention mean difference. For example, based on this example, the “team changes” component had a higher probability of ranking as one of the top 3 QI components, while “case management” appeared to rank in the bottom 3.

### Assessing nonadditivity 

**Figure 3:**
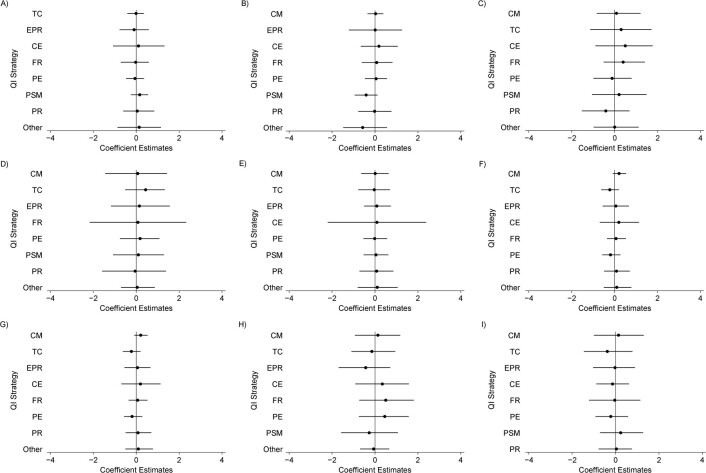
Coefficient estimates for pairwise product terms between quality improvement (QI) strategy components. The figure shows the results for pairwise product terms with A) case management (CM) (*n* = 65); B) team changes (TC) (*n* = 56); C) electronic patient registry (EPR) (*n* = 28); D) clinician education (CE) (*n* = 34); E) facilitated relay (FR) (*n* = 43); F) patient education (PE) (*n* = 109); G) promotion of self-management (PSM) (*n* = 93); H) patient reminders (PR) (*n* = 27); and I) “other” (*n* = 36). “Other” represents a combined category for infrequently evaluated components, including audit and feedback, clinician reminders, continuous quality improvement, and financial incentives. The following priors were used in the Bayesian analyses to estimate parameters in the models that included product terms: ${\mathrm{\beta}}_0\sim N\left(8,100\right)$; ${\mathrm{\beta}}_k\sim N\left(0,4\right)$; (the prior distributions for the mean of the product term γ) ${\mathrm{\gamma}}_l\sim N\left(0,2\right)$; and all $\mathrm{\tau} \sim U\left(0,2\right)$. For each model, the reported *n* indicates the number of observations (arms) for the component of interest (i.e., 65 arms in which CM was present).

Web Appendix 1 (equation 8) extends the model in equations 1 and 2 to include pairwise product terms (i.e., allowing for nonadditive associations) among QI components. A series of models were fitted that each included product terms for a single QI strategy, *r*, with all remaining QI strategies, $l\in \left\{1,\dots, m\right\}\backslash r$, corresponding to 8 additional parameters estimated in each of the 9 additional models (Web Appendix 6). We present results from these models in Figure 3. The CrIs of the coefficients of the product terms all crossed 0, consistent with the absence of nonadditive associations between the QI strategies. We therefore present results only from the more parsimonious models in the main paper.

### Assessing modification of associations by covariates

**Figure 4:**
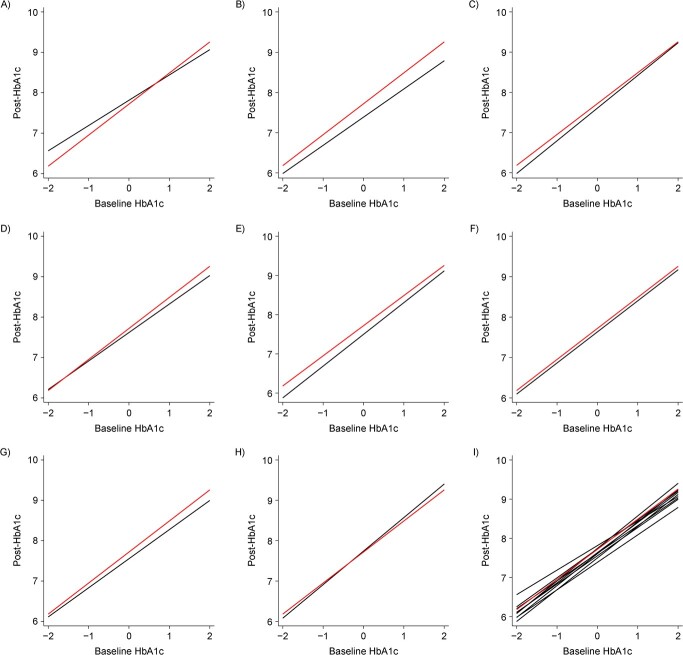
Modification of the association between quality improvement (QI) components and the posttreatment outcome mean by baseline hemoglobin A1c (HbA1c) level. A) Case management; B) team changes; C) electronic patient registry; D) clinician education; E) facilitated relay; F) patient education; G) promotion of self-management; H) patient reminders; I) all components combined. Baseline HbA1c values were centered. The red line shows the estimated posttreatment outcome mean without the QI strategy. The black line shows the estimated posttreatment outcome mean with the QI strategy. The following priors were used in the Bayesian analyses to estimate parameters in the association modification models: ${\mathrm{\beta}}_0\sim N\left(8,100\right)$; ${\mathrm{\beta}}_k\sim N\left(0,4\right)$; $\phi \sim N\left(0,4\right)$; ${\mathrm{\psi}}_k\sim N\left(0,4\right)$; and all $\mathrm{\tau} =U\left(0,2\right)$.

The original QI review identified the study average of HbA1c levels at baseline as a potential effect modifier. Thus, we explored the addition of product terms between baseline HbA1c (coded as both a binary and a continuous covariate) and each of the QI components as per equation 10 (Web Appendix 1). In the binary model, we used glycemic control of 8.0% to delineate between trials with patient populations that were “controlled” and “uncontrolled” at baseline. In the continuous model, we calculated a mean-centered HbA1c covariate. Both baseline HbA1c models included an additional 10 parameters (i.e., the coefficient of baseline HbA1c plus 9 coefficients for product terms between baseline HbA1c and QI strategies; see Web Appendices 7 and 8). Although estimates were not grossly incompatible with the absence of modification by HbA1c, there was some evidence that baseline HbA1c level modified the association between some QI strategies and the posttreatment mean outcome (Figure 4) and improved model predictions, particularly when treated as a continuous variable (see “Assessment of convergence, model evaluation, and robustness” section below). For example, the association between case management and the posttreatment mean outcome appeared to be greater when delivered in populations with higher baseline risk (Table 3). 1,2 However, the coefficients of the product terms were imprecisely estimated. In the end, because our results did not suggest systematic differences in posttreatment mean outcomes for different QI components over baseline HbA1c control, we continued to prefer our base model.

**Table 3:** Average Outcomes of Quality Improvement Strategies in Study Arms With a Controlled Hemoglobin A1c Level at Baseline Versus an Uncontrolled Level at Baseline

	**Uncontrolled HbA1c** ^**a**^ ^**,**^ ^**b**^ **+ QI Strategy (** ${\mathrm{\beta}}_0+{\mathrm{\beta}}_k+\phi +{\mathrm{\psi}}_k$ **)**		**Difference in Uncontrolled HbA1c** ^**a**^ **(** ${\mathrm{\beta}}_k+{\mathrm{\psi}}_k$ **)**		**Controlled HbA1c + QI Strategy** ^**a**^ ^**,**^ ^**c**^ **(** ${\mathrm{\beta}}_0+{\mathrm{\beta}}_k$ **)**		**Difference in Controlled HbA1c** ^**a**^ **(** ${\mathrm{\beta}}_k$ **)**		**Difference Between Differences** ^**a**^ $\left[{\boldsymbol{\mathrm{\beta}}}_{\boldsymbol{k}}-\left({\boldsymbol{\mathrm{\beta}}}_{\boldsymbol{k}}+{\boldsymbol{\mathrm{\psi}}}_{\boldsymbol{k}}\right)\right]$	
**QI Strategy**	**Median, % HbA1c**	**95% CrI**	**Median, % HbA1c**	**95% CrI**	**Median, % HbA1c**	**95% CrI**	**Median, % HbA1c**	**95% CrI**	**Median, % HbA1c**	**95% CrI**
CM	8.45	8.08, 8.82	−0.18	−0.48, 0.11	7.52	7.26, 7.80	0.12	−0.07, 0.33	0.30	−0.05, 0.67
TC	8.35	8.00, 8.72	−0.29	−0.56, 0.01	7.13	6.79, 7.46	−0.27	−0.56, 0.02	0.02	−0.41, 0.41
EPR	8.65	7.90, 9.34	0.01	−0.70, 0.66	7.32	6.98, 7.69	−0.08	−0.39, 0.26	−0.09	−0.78, 0.69
CE	8.53	7.84, 9.25	−0.11	−0.78, 0.59	7.30	7.00, 7.61	−0.10	−0.38, 0.20	0.01	−0.75, 0.75
FR	8.42	8.07, 8.79	−0.22	−0.51, 0.09	7.11	6.74, 7.50	−0.29	−0.64, 0.07	−0.08	−0.51, 0.40
PE	8.55	8.21, 8.89	−0.08	−0.40, 0.23	7.49	7.21, 7.76	0.09	−0.19, 0.36	0.18	−0.23, 0.58
PSM	8.45	8.09, 8.81	−0.19	−0.49, 0.11	7.13	6.78, 7.45	−0.27	−0.59, 0.01	−0.08	−0.51, 0.32
PR	8.75	8.12, 9.37	0.12	−0.48, 0.69	7.20	6.76, 7.61	−0.19	−0.62, 0.16	−0.31	−1.03, 0.36
Other^d^	8.48	7.66, 9.18	−0.15	−0.93, 0.49	7.42	7.11, 7.68	0.02	−0.25, 0.22	0.17	−0.52, 0.99

Abbreviations: CE, clinician education; CM, case management; CrI, credible interval; EPR, electronic patient registry; FR, facilitated relay; HbA1c, hemoglobin A1c; PE, patient education; PR, patient reminders; PSM, promotion of self-management; QI, quality improvement; TC, team changes.

^a^ The following priors were used in the Bayesian analyses to estimate parameters in the models allowing for modification of the association by HbA1c: ${\mathrm{\beta}}_0\sim N\left(8,100\right)$; ${\mathrm{\beta}}_k\sim N\left(0,4\right)$; $\phi \sim N\left(0,4\right)$; ${\mathrm{\psi}}_k\sim N\left(0,4\right)$; and all $\mathrm{\tau} \sim U\left(0,2\right)$.

^b^ Postintervention mean change in patients with an uncontrolled HbA1c level who did not receive the QI strategy: ${\mathrm{\beta}}_0+\phi$ = 8.63 (95% CrI: 8.42, 8.86).

^c^ Postintervention mean change in patients with a controlled baseline HbA1c level who did not receive the QI strategy: ${\mathrm{\beta}}_0$ = 7.40 (95% CrI: 7.24, 7.56).

^d^ “Other” represents a combined category for infrequently evaluated components, including audit and feedback, clinician reminders, continuous quality improvement, and financial incentives.

### Predicting outcomes for a novel combination of QI components

Assumingthat our response surface models are approximately correctly specified, we predict the distribution of the posttreatment mean for a specific combination of QI components in a new setting or trial (43). Table 4 presents the posterior predictive distribution of the outcome mean for novel combinations of components that were not observed in the included trials. For example, the QI components of “team changes,” “facilitated relay,” and “electronic patient registry” had relatively strong negative associations with the posttreatment outcome mean in analysis III and would be reasonable to combine in a novel intervention according to our content experts. The posterior and predictive distributions of the posttreatment mean (% HbA1c) in a new study with no QI components were estimated to be 8.14 (95% CrI: 7.96, 8.32) and 8.14 (95% CrI: 6.46, 9.81), respectively. We estimated that the new complex intervention would be associated with a substantially lower posttreatment mean. Using the posterior distribution, the posttreatment mean was 7.38% (95% CrI: 7.04, 7.72), and the mean difference as compared with no intervention was −0.75 (95% CrI: –1.05, −0.45). Using the posterior predictive distribution, the posttreatment mean was 7.38% (95% CrI: 5.56, 9.20), and the mean difference compared with no intervention was −0.75 (95% CrI: –1.51, −0.03).

**Table 4:** Estimated Median Values of the Posterior and Posterior Predictive Distributions of Average Outcomes for Untested Combinations of Quality Improvement Strategies

	**Posterior Distribution of the Posttreatment Mean**		**Difference From Baseline**		**Posterior Predictive Distribution of the Posttreatment Mean**		**Difference From Baseline**	
**Untested** ** Combination**	**Median, % HbA1c**	**95% CrI**	**Median, % HbA1c**	**95% CrI**	**Median, % HbA1c**	**95% CrI**	**Median, % HbA1c**	**95% CrI**
CM + EPR + FR	7.77	7.45, 8.09	−0.36	−0.64, −0.09	7.77	6.01, 9.53	−0.36	−0.94, 0.19
TC + FR + PSM	7.36	7.06, 7.66	−0.77	−1.03, −0.52	7.36	5.54, 9.18	−0.78	−1.50, −0.04
TC + FR + EPR	7.38	7.04, 7.72	−0.75	−1.05, −0.45	7.38	5.56, 9.20	−0.75	−1.51, −0.03

Abbreviations: CM, case management; CrI, credible interval; EPR, electronic patient registry; FR, facilitated relay; HbA1c, glycated hemoglobin; PSM, promotion of self-management; QI, quality improvement; TC, team changes.

### Assessment of convergence, model evaluation, and robustness

The upper limit of the 95% CrI for the Brooks-Gelman-Rubin statistic was less than 1.1 for 96% of all parameters monitored (and less than 1.2 for all parameters monitored).

**Figure 5:**
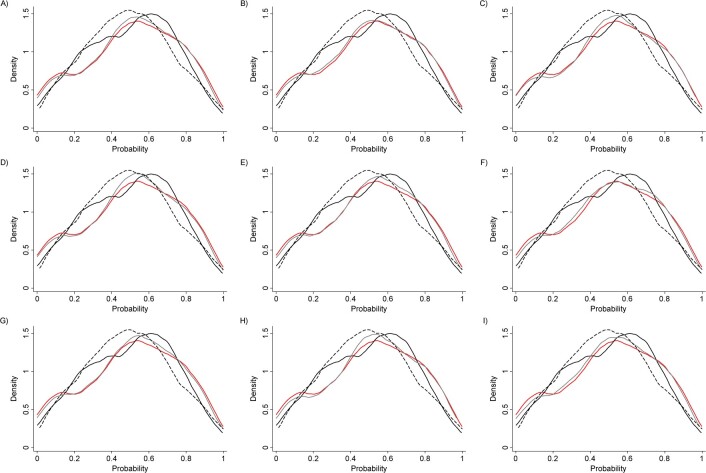
Posterior predictive checks. A) Quality improvement (QI) × case management; B) QI × team changes; C) QI × electronic patient registry; D) QI × clinician education; E) QI × facilitated relay; F) QI × patient education; G) QI × promotion of self-management; H) QI × patient reminders; I) QI × other. “Other” represents a combined category for infrequently evaluated components, including audit and feedback, clinician reminders, continuous quality improvement, and financial incentives. Each panel represents the posterior predictive check of the main model (red line), the model including product terms between QI components (9 models including a product term between one QI strategy and the other 8 QI strategies; gray line), the effect modification model when baseline hemoglobin A1c (HbA1c) level is treated as binary (black solid line), and the association modification model when baseline Hba1c is treated as continuous (black dashed line). These are densities of Bayesian 1-sided probabilities comparing observed and predicted mean outcomes. When the models fit well, one expects to see a mode at 0.5 (agreement of observed and predicted) with small mass at either extreme.

We used mixed posterior predictive checks (50) to compare the predicted outcome means from the models of new studies with the same combination of components as those observed in our sample against the observed outcome means. As illustrated in Figure 5, the meta-regression model performs reasonably well in terms of its probability of returning the observed means from parameter estimates. Other standard methods, such as the deviance information criterion, can also be used to evaluate model performance (50, 51). In general, the assessment approach should be chosen to reflect the goals of the modeling. We favor posterior predictive checks in our application because our primary goal is prediction. Because Bayesian posterior probabilities can be influenced by the specified priors (52), we performed sensitivity tests on our chosen priors and found that our findings were robust to these alterations.

Finally, we compared our analyses with meta-regressions that model differences in outcomes between study arms (53) rather than outcomes for each arm, and found similar estimates of differences in posttreatment mean outcomes for different QI components. Data for these analyses were sparser because the coefficients of components that are common in 2 or more arms of the same study “cancel” out, and thus the analyses took longer to converge (Web Table 3).

## DISCUSSION

We believe that *predicting* the average outcomes of novel complex interventions in new settings or future studies is a key goal when synthesizing evidence from trials of complex interventions. While any prediction model is unlikely to be correctly specified, examining a collection of models may offer useful insights for decision-making or planning future research: Patterns across evidence may be unearthed, and the diversity of the large data sets can be used as a strength rather than a limitation. Our experience is that decision-makers find these insights more useful than those afforded by conventional meta-analysis methods. The latter ask very abstract questions (e.g., doing something vs. nothing; including a component vs. not including it), do not adjust for combinations of components or study-level characteristics, and do not address data complications (e.g., missing data, differences in study designs).

Our approach includes all data from all studies and estimates the average posttreatment mean associated with each component. Our response surface models extrapolate to unobserved combinations of components and different settings by employing simplifying additivity assumptions. The assumptions are explicit and can be debated by substantive experts, examined statistically, and relaxed by using more flexible models if the data permit. Natural model extensions can handle missing data or other complications that arise in evidence synthesis of trials of complex interventions. We focused on models that use arm-level data, though models based on differences are also possible and led to similar results in our example.

We have no hope of observing empirical data on all 4,095 nonempty combinations of 12 components; we must extrapolate from the few observed combinations, about 2% of the total, to the remaining 98% of unobserved ones through a model which we argue lends itself to a response surface approach. The models in equations 1–4 enable these extrapolations while reducing the parameter space from order ${2}^m$, in a fully saturated nonparametric model, to an order *m*, in a model of main effects only. This 3-orders-of-magnitude reduction in the number of parameters assumes that the information modeled by the omitted parameters is negligible. However, making such assumptions and testing some of them in alternative models (e.g., models that add pairwise interactions between components) allows us to acquire *workin*g *predictions* that can be used by policy-makers and researchers who plan future research. Working predictions are useful: Policy-makers must make decisions even without data—and in many cases, their initial decisions are tentative, dynamically monitored, and subject to revision. Researchers who plan novel interventions will eventually put them to the test in randomized experiments or through observation. This viewpoint builds on a conceptual proposal that meta-analysis is best viewed as response surface estimation (25), and is most similar to the application of evidence synthesis in social science (24).

All predictions are, of course, conditional on the model, and the “true” model is ultimately unknown. Substantial heterogeneity in results will often remain in applications of our approach, because the representation of a complex intervention as a vector of components does not capture everything about an intervention (54–56); the measured study level attributes do not capture everything about populations and settings; and important covariates are poorly measured or missing. These difficulties limit the potential usefulness of predictive modeling based on aggregate data (57, 58) and are perhaps even more limiting for analyses aspiring to produce results that have causal interpretations.

In conclusion, predicting the average outcomes of complex interventions in a new setting or study is a key goal for evidence synthesis of trials comparing complex interventions. Collections of meta-regression models can be used to estimate the response surface relating study outcomes to intervention components and study characteristics, to isolate component-specific associations with outcomes, and to predict the outcomes of complex interventions (including those not previously evaluated) in new populations or settings, while addressing common data complications.

## Supplementary Material

Web_Material_kwad184
