# Supplementary material for: Evidence Synthesis for Complex Interventions Using Meta-Regression Models
Source: Am J Epidemiol. 2023 Sep 8;193(2):323–38. doi: 10.1093/aje/kwad184 (PMC10840082; doi:10.1093/aje/kwad184)
Supplement: Web_Material_kwad184 [file web_material_kwad184.zip › kwad184 Konnyu Web Material.pdf]

WEB MATERIAL

Evidence Synthesis for Complex Interventions Using Meta-Regression Models

K. Konnyu, J. M. Grimshaw, T. A. Trikalinos, N. M. Ivers, D. Moher, and I. J. Dahabreh

Table of Contents

WEB APPENDIX 1. MODEL EXTENSIONS 2

WEB APPENDIX 2. IMPUTATION OF MISSING VARIANCES AND ESTIMATES OF THE INTRACLASS CORRELATION COEFFICIENT 5

WEB APPENDIX 3. GROUPS OF STUDIES FOR HIERARCHICAL MODELS 6

WEB TABLE 1. ANALYSIS BINS FOR ANALYSIS II (TWO-ARMS: MOST INTENSIVE VERSUS LEAST INTENSIVE) 6

WEB TABLE 2. ANALYSIS III (ALL ARMS) 6

WEB APPENDIX 4. META-REGRESSION MODEL IMPLEMENTED IN ANALYSIS II 8

WEB APPENDIX 5. META-REGRESSION MODEL IMPLEMENTED IN ANALYSIS III (BASE MODEL) 10

WEB APPENDIX 6. META-REGRESSION MODEL EXTENSION: INTERACTIONS AMONG INTERVENTION COMPONENTS 12

WEB APPENDIX 7. META-REGRESSION MODEL EXTENSION: EFFECT MODIFICATION – BINARY HBA1C 15

WEB APPENDIX 8. META-REGRESSION MODEL EXTENSION: EFFECT MODIFICATION – CONTINUOUS HBA1C 18

WEB TABLE 3. SUMMARY OF RESULTS COMPARING META-REGRESSION OF STUDY-LEVEL DIFFERENCES VS. RESPONSE SURFACE META-REGRESSION OF ARM MEANS 22

REFERENCES FOR WEB APPENDIX 23

Web Appendix 1. Model Extensions

In this appendix we extend the basic model presented in the main text ("Specification of the basic response surface meta-regression model and of prior distributions and inference") to relax the additivity assumptions of Equation 2, specify models for discrete outcomes, and handle missing data. We use the term interaction to describe the differential effect of one intervention component depending on the presence or absence of another intervention component; we use the term effect modification to describe the differential effect of one intervention component over levels of a baseline study-level covariate (e.g., setting of care, baseline outcome risk, population age).

Interactions among intervention components

We can assess interactions by including product terms among intervention components (1, 2). For example, to allow for interaction of component r with intervention component l, we might specify the mean model

$$\mu_{ij} = \beta_{0i} + \sum_{k=1}^{m} \beta_{ki} X_{ijk} + \sum_{l \in \{1,\ldots,m\} \setminus r} \gamma_{li} X_{ijl} X_{ijr} \, , \tag{8}$$

where $\{1, \ldots, m\} \setminus r$ denotes that summation is over all components except r. We assume that the interaction coefficients γ_{li} are distributed as

$$\gamma_{li} \sim N(\gamma_l, \tau_l^2), \qquad l \in \{1, \ldots, m\} \setminus r. \tag{9}$$

For prior distributions we used the minimally informative normal and uniform distributions for γ_l and τ_l (see the Discussion section in the main paper).

In most applications, we do not have data to assess all hypothesized interactions or effect modifiers of interest. For example, for m intervention components, there are $m(m-1)/2$ possible pairwise interactions. In the case of the diabetes QI review, this equates to 66 pairwise interactions formed from 12 QI components. As discussed, available studies are unlikely to provide sufficient data to support a fully saturated interaction model (i.e., one that would include all possible interactions, up to the 12-way interaction of all components) – such a model would have enough coefficients to (non-parametrically) model 4,095 QI combinations, which is equivalent to a network meta-analysis where each possible combination of components is treated as a separate treatment node. To obtain reasonable results, meta-analysts will need to make decisions as to what interactions to pursue, within the limitations of available data (3). Depending on the number of intervention components, reviewers may consider model reduction strategies such as aggregating intervention components into higher order categories based on substantive knowledge and/or expert opinion or grouping uncommon interventions into a single 'mixed/other' category (4).

Effect modification by study covariates

The model can also be extended to assess effect modification by study-level covariates, such as study setting or other contextual factors, with the addition of appropriate product terms like those described in the main manuscript (1). For example, let Z_i denote an indicator for whether the

study was conducted in a for-profit or not-for-profit setting; then, we might use the post-treatment mean model:

$$\mu_{ij} = \beta_{0i} + \sum_{k=1}^{m} \beta_{ki} X_{ijk} + \phi_i Z_i + \sum_{k=1}^{m} \psi_{ki} Z_i X_{ijk} \tag{10}$$

where ϕ_i is the "main effect" of the modifier and ψ_{ki} is the "statistical" interaction coefficient of the modifier with the kth intervention component. We assume the ϕ_i are distributed as

$$\phi_i \sim N(\phi, \tau_\phi^2) \tag{11}$$

and the ψ_i are distributed as

$$\psi_{ki} \sim N(\psi_k, \tau_{\psi k}^2). \tag{12}$$

Again, in Bayesian analyses, prior distributions need to be specified, and we chose the minimally informative distributions for both ($\phi \sim N(0,4)$ and $\tau_\phi \sim U(0,2)$; $\psi_k \sim N(0,4)$ and $\tau_{\psi k} \sim U(0,2)$. Most applied meta-analyses of complex interventions will not have sufficient data to allow joint examination of all possible effect modifiers, and meta-analysts will need to decide what effect modifiers to pursue based on substantive knowledge.

Discrete outcomes

The model can be modified to assess the impact of complex interventions on non-linear and non-continuous outcomes (e.g., binary, count) by replacing Equations 1 and 2 with the appropriate link functions and likelihood for the response variable, respectively (5). For example, for binary response data (e.g., proportion of patients on anti-hypertensive medication), Equation 1 is modified such that the count of events in the jth arm of the ith study is a realization of Y_{ij}, a binomial random variable,

$$Y_{ij} \sim Bin(n_{ij}, p_{ij}), \tag{12}$$

where n_{ij} is the number of individuals and p_{ij} is the probability of event occurrence in the jth arm of the ith study. A common choice for the mean model is a logistic-linear specification,

$$\text{logit}(p_{ij}) = \beta_{0i} + \sum_{k=1}^{m} \beta_{ki} X_{ijk}. \tag{13}$$

Missing data

Data are often missing from studies (e.g., post outcome mean and its variance) due to incomplete (and possibly selective) reporting (6). Meta-analysts will often impute missing data to allow for the synthesis of data from the greatest possible proportion (if not all) of included studies for that outcome (e.g., replace missing standard deviation with the median of reported standard deviations of included studies) (6). A meta-regression model implemented in a Bayesian framework provides a convenient structure to impute missing data. In the application, the standard error of the post-treatment mean was missing from 68 arms of patient randomized trials

and 31 arms of cluster randomized trials. In addition, 49 standard errors of cluster randomized trials were unadjusted, that is, they did not account for the non-independence of observed patient outcomes due to the cluster randomized design (or report an estimate of the ICC to correct unadjusted standard errors). Therefore, to include data from both individually randomized and cluster randomized trials, we needed to impute missing standard errors and ICCs, where relevant. Details on our approach are provided in **Web Appendix 2**. Briefly, using expert opinion combined with information from the QI intervention review, we imputed missing standard errors from a moderately informative uniform distribution $U(0,2)$. We imputed missing ICCs from a posterior predictive distribution obtained from the synthesis of ICCs in the diabetes QI review and external sources, using an approach recommended by Turner et al. (7) and described elsewhere (8).

Web Appendix 2. Imputation of Missing Variances and Estimates of the Intraclass Correlation Coefficient

Missing estimates of variance

- In extraction, we sought the standard deviation or standard error of post-intervention group mean.
- If unavailable, we extracted other measures of variance and calculated the standard error according to established methods. (9, 10)
- For meta-analyses, we converted extracted standard deviations to sample standard errors
- After performing the above, there was missing data for 68 observations (standard errors of group means) from patient randomized trials and missing data from 31 observations from cluster randomized trials (99 of 241 observations; 41%).
- The median and range for observed standard errors was 0.18 (0.08-0.51). We opted to impute missing standard errors from a uniform distribution from 0 to 2.

Missing estimates of ICC

- There were 23 cluster randomized trials that assessed HbA1c in the example dataset.
- Of these, no study reported adjusted standard errors of the post group means of HbA1c.
- Two studies (included one excluded from the analyses because it did not report baseline HbA1c) reported estimates of the intraclass correlation coefficient (ICC). We used these ICC to calculate a correction factor to adjust reported standard errors for those two studies.
- The remainder of standard errors from cluster randomized trials (corresponding to 49 observations) required adjustment from an external ICC estimate.
- We opted to impute missing ICCs from a predictive distribution obtained from the synthesis of ICCs in the diabetes QI review and external sources. Based on 9 estimates of the ICC for the HbA1c outcome, we estimated a posterior predictive distribution with a logit-transformed mean of -4.16224 and standard deviation of 1.8812.

Web Appendix 3. Groups of Studies for Hierarchical Models

The models presented in **Web Appendices 4** and **5** applied the same linear model to different analysis bins. In parsing data this way, we were able to account for nuanced design and reporting challenges apparent in our sample of included studies, including missing variances and ICCs. The following tables describe the content of the analysis bins, the number of studies and number of arms, and how data within each bin were handled uniquely. Data from Analysis III was used for all subsequent meta-regression models described in the main paper.

Web Table 1. Analysis Bins for Analysis II (Two-arms: Most Intensive versus Least Intensive)

**Analysis bin**	**Data characteristics**	**Arms (n=228)[a]**	**Studies (n=114)**	**Model parsing**
1	Patient RCT, variance reported<br>Run model	118	59	1-59
2	Cluster RCT, variance reported, cluster adjusted<br>Run model	0	0	
3	Cluster RCT, variance reported, not cluster adjusted, ICC reported<br>Adjust with ICC, then run model	0	0	--
4	Cluster RCT, variance reported, not cluster adjusted, no ICC reported<br>Impute ICC, adjust with ICC, then run model	18	9	60-68
5	Patient RCT, no variance reported<br>Impute SE, then run model	64	32	69-100
6	Cluster RCT, no variance reported, not cluster adjusted, ICC reported<br>Impute SE, adjust with ICC, then run model	2	1	101
7	Cluster RCT, no variance reported, not cluster adjusted, no ICC reported<br>Impute SE, impute ICC, adjust with ICC, then run model	26	13	102-114

Abbreviations: ICC=intracluster correlation coefficient, RCT=randomized controlled trial, SE=standard error

[a]13 arms from 8 multiarm studies were dropped

Web Table 2. Analysis III (All Arms)

**Analysis bin**	**Data characteristics**	**Arms (n=241)**	**Studies (n=114)**	**Model parsing**
1	Patient RCT, variance reported<br>Run model	122	59	1-59
2	Cluster RCT, variance reported, cluster adjusted<br>Run model	0	0	
3	Cluster RCT, variance reported, not cluster adjusted, ICC reported	0	0	--

	Adjust with ICC, then run model			
4	Cluster RCT, variance reported, not cluster adjusted, no ICC reported<br>Impute ICC, adjust with ICC, then run model	20	9	60-68
5	Patient RCT, no variance reported<br>Impute SE, then run model	68	32	69-100
6	Cluster RCT, no variance reported, not cluster adjusted, ICC reported<br>Impute SE, adjust with ICC, then run model	2	1	101-101
7	Cluster RCT, no variance reported, not cluster adjusted, no ICC reported<br>Impute SE, impute ICC, adjust with ICC, then run model	29	13	102-114

Abbreviations: ICC=intracluster correlation coefficient, RCT=randomized controlled trial, SE=standard error

Web Appendix 4. Meta-Regression Model Implemented in Analysis II

```
model {
        for(i in 1:59) {
                for(j in 1:n_arms[i]) {
                        y[i,j] ~ dnorm(mu[i,j] , prec.resp[i,j])
                        mu[i,j] = beta[i,1] + beta[i,2] * CM[i,j]
                                                                + beta[i,3] * TC[i,j]
                                                                + beta[i,4] * EPR[i,j]
                                                                + beta[i,5] * CE[i,j]
                                                                + beta[i,6] * FR[i,j]
                                                                + beta[i,7] * PE[i,j]
                                                                + beta[i,8] * PSM[i,j]
                                                                + beta[i,9] * PR[i,j]
                                                                + beta[i,10] * Other[i,j]
                        prec.resp[i,j] <- (1/se[i,j])*(1/se[i,j])
                        }
                for(m in 1:10) {
                beta[i,m] ~ dnorm(mu_bar[m], prec[m])
                }
        }
for(i in 60:68) {
                for(j in 1:n_arms[i]) {
                        y[i,j] ~ dnorm(mu[i,j] , prec.resp[i,j])
                        mu[i,j] = beta[i,1] + beta[i,2] * CM[i,j]
                                                                + beta[i,3] * TC[i,j]
                                                                + beta[i,4] * EPR[i,j]
                                                                + beta[i,5] * CE[i,j]
                                                                + beta[i,6] * FR[i,j]
                                                                + beta[i,7] * PE[i,j]
                                                                + beta[i,8] * PSM[i,j]
                                                                + beta[i,9] * PR[i,j]
                                                                + beta[i,10] * Other[i,j]
                        prec.resp[i,j] <- (1/(se[i,j]*sqrt(corrf[i,j])))*(1/(se[i,j]*sqrt(corrf[i,j])))
                        corrf[i,j] <- 1 + (avg_cluster_size[i,j]-1) * ICC[i,j]
                        ICC[i,j] <- exp(logit_ICC[i,j]) / ( 1 + exp(logit_ICC[i,j]))
                        logit_ICC[i,j] ~ dnorm(-4.16224, (1/(1.8812*1.8812)))
                        }
                for(m in 1:10) {
                beta[i,m] ~ dnorm(mu_bar[m], prec[m])
                }
        }
for(i in 69:100) {
                for(j in 1:n_arms[i]) {
                        y[i,j] ~ dnorm(mu[i,j] , prec.resp[i,j])
                        mu[i,j] = beta[i,1] + beta[i,2] * CM[i,j]
                                                                + beta[i,3] * TC[i,j]
                                                                + beta[i,4] * EPR[i,j]
                                                                + beta[i,5] * CE[i,j]
                                                                + beta[i,6] * FR[i,j]
                                                                + beta[i,7] * PE[i,j]
                                                                + beta[i,8] * PSM[i,j]
                                                                + beta[i,9] * PR[i,j]
                                                                + beta[i,10] * Other[i,j]
                        prec.resp[i,j] <- (1/se[i,j])*(1/se[i,j])
```

```
                        se[i,j] ~ dunif(0,2)
                        }
                for(m in 1:10) {
                beta[i,m] ~ dnorm(mu_bar[m], prec[m])
                }
        }
for(i in 101:101) {
                for(j in 1:n_arms[i]) {
                        y[i,j] ~ dnorm(mu[i,j] , prec.resp[i,j])
                        mu[i,j] = beta[i,1] + beta[i,2] * CM[i,j]
                                                                + beta[i,3] * TC[i,j]
                                                                + beta[i,4] * EPR[i,j]
                                                                + beta[i,5] * CE[i,j]
                                                                + beta[i,6] * FR[i,j]
                                                                + beta[i,7] * PE[i,j]
                                                                + beta[i,8] * PSM[i,j]
                                                                + beta[i,9] * PR[i,j]
                                                                + beta[i,10] * Other[i,j]
                        se[i,j] ~ dunif(0, 2)
                        prec.resp[i,j] <- (1/(se[i,j]*sqrt(corrf[i,j])))*(1/(se[i,j]*sqrt(corrf[i,j])))
                        corrf[i,j] <- 1 + (avg_cluster_size[i,j]-1) * ICC[i,j]
                        }
                for(m in 1:10) {
                beta[i,m] ~ dnorm(mu_bar[m], prec[m])
                }
        }
for(i in 102:114) {
                for(j in 1:n_arms[i]) {
                        y[i,j] ~ dnorm(mu[i,j] , prec.resp[i,j])
                        mu[i,j] = beta[i,1] + beta[i,2] * CM[i,j]
                                                                + beta[i,3] * TC[i,j]
                                                                + beta[i,4] * EPR[i,j]
                                                                + beta[i,5] * CE[i,j]
                                                                + beta[i,6] * FR[i,j]
                                                                + beta[i,7] * PE[i,j]
                                                                + beta[i,8] * PSM[i,j]
                                                                + beta[i,9] * PR[i,j]
                                                                + beta[i,10] * Other[i,j]
                        se[i,j] ~ dunif(0,2)
                        prec.resp[i,j] <- (1/(se[i,j]*sqrt(corrf[i,j])))*(1/(se[i,j]*sqrt(corrf[i,j])))
                        corrf[i,j] <- 1 + (avg_cluster_size[i,j]-1) * ICC[i,j]
                        ICC[i,j] <- exp(logit_ICC[i,j]) / ( 1 + exp(logit_ICC[i,j]))
                        logit_ICC[i,j] ~ dnorm(-4.16224, (1/(1.8812*1.8812)))
                        }
                for(m in 1:10) {
                beta[i,m] ~ dnorm(mu_bar[m], prec[m])
                }
        }
                mu_bar[1] ~ dnorm(8,0.01)
                for(m in 2:10) {   mu_bar[m] ~ dnorm(0,0.25) }
                for(m in 1:10) {
                                prec[m] = (1/tau[m]) * (1/tau[m])
                                }
                tau[1] ~ dunif(0,2)
                for(m in 2:10) {   tau[m] ~ dunif(0,2) }
}
```

}

Web Appendix 5. Meta-Regression Model Implemented in Analysis III (Base model)

```
model {
	for(i in 1:59) {
		for(j in 1:n_arms[i]) {
			y[i,j] ~ dnorm(mu[i,j] , prec.resp[i,j])
			mu[i,j] = beta[i,1] + beta[i,2] * CM[i,j]
							+ beta[i,3] * TC[i,j]
							+ beta[i,4] * EPR[i,j]
							+ beta[i,5] * CE[i,j]
							+ beta[i,6] * FR[i,j]
							+ beta[i,7] * PE[i,j]
							+ beta[i,8] * PSM[i,j]
							+ beta[i,9] * PR[i,j]
							+ beta[i,10] * Other[i,j]
			prec.resp[i,j] <- (1/se[i,j])*(1/se[i,j])
			}
		for(m in 1:10) {
		beta[i,m] ~ dnorm(mu_bar[m], prec[m])
		}
	}
for(i in 60:68) {
		for(j in 1:n_arms[i]) {
			y[i,j] ~ dnorm(mu[i,j] , prec.resp[i,j])
			mu[i,j] = beta[i,1] + beta[i,2] * CM[i,j]
							+ beta[i,3] * TC[i,j]
							+ beta[i,4] * EPR[i,j]
							+ beta[i,5] * CE[i,j]
							+ beta[i,6] * FR[i,j]
							+ beta[i,7] * PE[i,j]
							+ beta[i,8] * PSM[i,j]
							+ beta[i,9] * PR[i,j]
							+ beta[i,10] * Other[i,j]
			prec.resp[i,j] <- (1/(se[i,j]*sqrt(corrf[i,j])))*(1/(se[i,j]*sqrt(corrf[i,j])))
			corrf[i,j] <- 1 + (avg_cluster_size[i,j]-1) * ICC[i,j]
			ICC[i,j] <- exp(logit_ICC[i,j]) / ( 1 + exp(logit_ICC[i,j]))
			logit_ICC[i,j] ~ dnorm(-4.16224, (1/(1.8812*1.8812)))
			}
		for(m in 1:10) {
		beta[i,m] ~ dnorm(mu_bar[m], prec[m])
		}
	}
for(i in 69:100) {
		for(j in 1:n_arms[i]) {
			y[i,j] ~ dnorm(mu[i,j] , prec.resp[i,j])
			mu[i,j] = beta[i,1] + beta[i,2] * CM[i,j]
							+ beta[i,3] * TC[i,j]
							+ beta[i,4] * EPR[i,j]
							+ beta[i,5] * CE[i,j]
							+ beta[i,6] * FR[i,j]
							+ beta[i,7] * PE[i,j]
							+ beta[i,8] * PSM[i,j]
							+ beta[i,9] * PR[i,j]
							+ beta[i,10] * Other[i,j]
			prec.resp[i,j] <- (1/se[i,j])*(1/se[i,j])
			se[i,j] ~ dunif(0,2)
			}
		for(m in 1:10) {
		beta[i,m] ~ dnorm(mu_bar[m], prec[m])
		}
	}
```

```
for(i in 101:101) {
		for(j in 1:n_arms[i]) {
			y[i,j] ~ dnorm(mu[i,j] , prec.resp[i,j])
			mu[i,j] = beta[i,1] + beta[i,2] * CM[i,j]
								+ beta[i,3] * TC[i,j]
								+ beta[i,4] * EPR[i,j]
								+ beta[i,5] * CE[i,j]
								+ beta[i,6] * FR[i,j]
								+ beta[i,7] * PE[i,j]
								+ beta[i,8] * PSM[i,j]
								+ beta[i,9] * PR[i,j]
								+ beta[i,10] * Other[i,j]
			se[i,j] ~ dunif(0, 2)
			prec.resp[i,j] <- (1/(se[i,j]*sqrt(corrf[i,j])))*(1/(se[i,j]*sqrt(corrf[i,j])))
			corrf[i,j] <- 1 + (avg_cluster_size[i,j]-1) * ICC[i,j]
			}
		for(m in 1:10) {
		beta[i,m] ~ dnorm(mu_bar[m], prec[m])
		}
	}
for(i in 102:114) {
		for(j in 1:n_arms[i]) {
			y[i,j] ~ dnorm(mu[i,j] , prec.resp[i,j])
			mu[i,j] = beta[i,1] + beta[i,2] * CM[i,j]
								+ beta[i,3] * TC[i,j]
								+ beta[i,4] * EPR[i,j]
								+ beta[i,5] * CE[i,j]
								+ beta[i,6] * FR[i,j]
								+ beta[i,7] * PE[i,j]
								+ beta[i,8] * PSM[i,j]
								+ beta[i,9] * PR[i,j]
								+ beta[i,10] * Other[i,j]
			se[i,j] ~ dunif(0,2)
			prec.resp[i,j] <- (1/(se[i,j]*sqrt(corrf[i,j])))*(1/(se[i,j]*sqrt(corrf[i,j])))
			corrf[i,j] <- 1 + (avg_cluster_size[i,j]-1) * ICC[i,j]
			ICC[i,j] <- exp(logit_ICC[i,j]) / ( 1 + exp(logit_ICC[i,j]))
			logit_ICC[i,j] ~ dnorm(-4.16224, (1/(1.8812*1.8812)))
			}
		for(m in 1:10) {
		beta[i,m] ~ dnorm(mu_bar[m], prec[m])
		}
	}
		mu_bar[1] ~ dnorm(8,0.01)
		for(m in 2:10) {	mu_bar[m] ~ dnorm(0,0.25) }
		for(m in 1:10) {
				prec[m] = (1/tau[m]) * (1/tau[m])
				}
		tau[1] ~ dunif(0,2)
		for(m in 2:10) {	tau[m] ~ dunif(0,2) }
		rk <-rank(mu_bar[2:10])
for(m in 1:10) {
	mu_bar_new[m] ~ dnorm(mu_bar[m] , prec[m])
}
```

Web Appendix 6. Meta-Regression Model Extension: Interactions Among Intervention Components

NB: We ran 9 versions of this model representing pairwise interactions between a QI strategy and each of the other QI strategies. Presented below is the pairwise interaction model of case management (CM) with all other QI strategies.

```
model {
        for(i in 1:59) {
                for(j in 1:n_arms[i]) {
                        y[i,j] ~ dnorm(mu[i,j] , prec.resp[i,j])
                        mu[i,j] = beta[i,1] + beta[i,2] * CM[i,j]
                                        + beta[i,3] * TC[i,j]
                                        + beta[i,4] * EPR[i,j]
                                        + beta[i,5] * CE[i,j]
                                        + beta[i,6] * FR[i,j]
                                        + beta[i,7] * PE[i,j]
                                        + beta[i,8] * PSM[i,j]
                                        + beta[i,9] * PR[i,j]
                                        + beta[i,10] * Other[i,j]
                                        + beta[i,11] * TC[i,j] * CM[i,j]
                                        + beta[i,12] * EPR[i,j] * CM[i,j]
                                        + beta[i,13] * CE[i,j] * CM[i,j]
                                        + beta[i,14] * FR[i,j] * CM[i,j]
                                        + beta[i,15] * PE[i,j] * CM[i,j]
                                        + beta[i,16] * PSM[i,j] * CM[i,j]
                                        + beta[i,17] * PR[i,j] * CM[i,j]
                                        + beta[i,18] * Other[i,j] * CM[i,j]
                        prec.resp[i,j] <- (1/se[i,j])*(1/se[i,j])
                        }
                for(m in 1:18) {
                beta[i,m] ~ dnorm(mu_bar[m], prec[m])
                }
        }
for(i in 60:68) {
                for(j in 1:n_arms[i]) {
                        y[i,j] ~ dnorm(mu[i,j] , prec.resp[i,j])
                        mu[i,j] = beta[i,1] + beta[i,2] * CM[i,j]
                                        + beta[i,3] * TC[i,j]
                                        + beta[i,4] * EPR[i,j]
                                        + beta[i,5] * CE[i,j]
                                        + beta[i,6] * FR[i,j]
                                        + beta[i,7] * PE[i,j]
                                        + beta[i,8] * PSM[i,j]
                                        + beta[i,9] * PR[i,j]
                                        + beta[i,10] * Other[i,j]
                                        + beta[i,11] * TC[i,j] * CM[i,j]
                                        + beta[i,12] * EPR[i,j] * CM[i,j]
                                        + beta[i,13] * CE[i,j] * CM[i,j]
                                        + beta[i,14] * FR[i,j] * CM[i,j]
                                        + beta[i,15] * PE[i,j] * CM[i,j]
                                        + beta[i,16] * PSM[i,j] * CM[i,j]
                                        + beta[i,17] * PR[i,j] * CM[i,j]
                                        + beta[i,18] * Other[i,j] * CM[i,j]
                        prec.resp[i,j] <- (1/(se[i,j]*sqrt(corrf[i,j])))*(1/(se[i,j]*sqrt(corrf[i,j])))
                        corrf[i,j] <- 1 + (avg_cluster_size[i,j]-1) * ICC[i,j]
                        ICC[i,j] <- exp(logit_ICC[i,j]) / ( 1 + exp(logit_ICC[i,j]))
                        logit_ICC[i,j] ~ dnorm(-4.16224, (1/(1.8812*1.8812)))
                        }
                for(m in 1:18) {
                beta[i,m] ~ dnorm(mu_bar[m], prec[m])
                }
```

```
	}
for(i in 69:100) {
		for(j in 1:n_arms[i]) {
			y[i,j] ~ dnorm(mu[i,j] , prec.resp[i,j])
			mu[i,j] = beta[i,1] + beta[i,2] * CM[i,j]
							+ beta[i,3] * TC[i,j]
							+ beta[i,4] * EPR[i,j]
							+ beta[i,5] * CE[i,j]
							+ beta[i,6] * FR[i,j]
							+ beta[i,7] * PE[i,j]
							+ beta[i,8] * PSM[i,j]
							+ beta[i,9] * PR[i,j]
							+ beta[i,10] * Other[i,j]
							+ beta[i,11] * TC[i,j] * CM[i,j]
							+ beta[i,12] * EPR[i,j] * CM[i,j]
							+ beta[i,13] * CE[i,j] * CM[i,j]
							+ beta[i,14] * FR[i,j] * CM[i,j]
							+ beta[i,15] * PE[i,j] * CM[i,j]
							+ beta[i,16] * PSM[i,j] * CM[i,j]
							+ beta[i,17] * PR[i,j] * CM[i,j]
							+ beta[i,18] * Other[i,j] * CM[i,j]
			prec.resp[i,j] <- (1/se[i,j])*(1/se[i,j])
			se[i,j] ~ dunif(0,2)
			}
		for(m in 1:18) {
		beta[i,m] ~ dnorm(mu_bar[m], prec[m])
		}
	}
for(i in 101:101) {
		for(j in 1:n_arms[i]) {
			y[i,j] ~ dnorm(mu[i,j] , prec.resp[i,j])
			mu[i,j] = beta[i,1] + beta[i,2] * CM[i,j]
							+ beta[i,3] * TC[i,j]
							+ beta[i,4] * EPR[i,j]
							+ beta[i,5] * CE[i,j]
							+ beta[i,6] * FR[i,j]
							+ beta[i,7] * PE[i,j]
							+ beta[i,8] * PSM[i,j]
							+ beta[i,9] * PR[i,j]
							+ beta[i,10] * Other[i,j]
							+ beta[i,11] * TC[i,j] * CM[i,j]
							+ beta[i,12] * EPR[i,j] * CM[i,j]
							+ beta[i,13] * CE[i,j] * CM[i,j]
							+ beta[i,14] * FR[i,j] * CM[i,j]
							+ beta[i,15] * PE[i,j] * CM[i,j]
							+ beta[i,16] * PSM[i,j] * CM[i,j]
							+ beta[i,17] * PR[i,j] * CM[i,j]
							+ beta[i,18] * Other[i,j] * CM[i,j]
			se[i,j] ~ dunif(0, 2)
			prec.resp[i,j] <- (1/(se[i,j]*sqrt(corrf[i,j])))*(1/(se[i,j]*sqrt(corrf[i,j])))
			corrf[i,j] <- 1 + (avg_cluster_size[i,j]-1) * ICC[i,j]
			}
		for(m in 1:18) {
		beta[i,m] ~ dnorm(mu_bar[m], prec[m])
		}
	}
for(i in 102:114) {
		for(j in 1:n_arms[i]) {
			y[i,j] ~ dnorm(mu[i,j] , prec.resp[i,j])
			mu[i,j] = beta[i,1] + beta[i,2] * CM[i,j]
							+ beta[i,3] * TC[i,j]
							+ beta[i,4] * EPR[i,j]
```

```
                                                    + beta[i,5] * CE[i,j]
                                                    + beta[i,6] * FR[i,j]
                                                    + beta[i,7] * PE[i,j]
                                                    + beta[i,8] * PSM[i,j]
                                                    + beta[i,9] * PR[i,j]
                                                    + beta[i,10] * Other[i,j]
                                                    + beta[i,11] * TC[i,j] * CM[i,j]
                                                    + beta[i,12] * EPR[i,j] * CM[i,j]
                                                    + beta[i,13] * CE[i,j] * CM[i,j]
                                                    + beta[i,14] * FR[i,j] * CM[i,j]
                                                    + beta[i,15] * PE[i,j] * CM[i,j]
                                                    + beta[i,16] * PSM[i,j] * CM[i,j]
                                                    + beta[i,17] * PR[i,j] * CM[i,j]
                                                    + beta[i,18] * Other[i,j] * CM[i,j]
                        se[i,j] ~ dunif(0,2)
                        prec.resp[i,j] <- (1/(se[i,j]*sqrt(corrf[i,j])))*(1/(se[i,j]*sqrt(corrf[i,j])))
                        corrf[i,j] <- 1 + (avg_cluster_size[i,j]-1) * ICC[i,j]
                        ICC[i,j] <- exp(logit_ICC[i,j]) / ( 1 + exp(logit_ICC[i,j]))
                        logit_ICC[i,j] ~ dnorm(-4.16224, (1/(1.8812*1.8812)))
                        }
                for(m in 1:18) {
                beta[i,m] ~ dnorm(mu_bar[m], prec[m])
                }
        }

                mu_bar[1] ~ dnorm(8,0.01)
                for(m in 2:10) {        mu_bar[m] ~ dnorm(0,0.25) }
                for(m in 11:18) {       mu_bar[m] ~ dnorm(0,0.50) }
                for(m in 1:18) {
                                        prec[m] = (1/tau[m]) * (1/tau[m])
                                        }
                tau[1] ~ dunif(0,2)
                for(m in 2:18) {        tau[m] ~ dunif(0,2) }
for(m in 1:18) {
        mu_bar_new[m] ~ dnorm(mu_bar[m] , prec[m])
}
}
```

Web Appendix 7. Meta-Regression Model Extension: Effect Modification – Binary HbA1c

```
model {
        for(i in 1:59) {
                for(j in 1:n_arms[i]) {
                        y[i,j] ~ dnorm(mu[i,j] , prec.resp[i,j])
                        mu[i,j] = beta[i,1] + beta[i,2] * CM[i,j]
                                        + beta[i,3] * TC[i,j]
                                        + beta[i,4] * EPR[i,j]
                                        + beta[i,5] * CE[i,j]
                                        + beta[i,6] * FR[i,j]
                                        + beta[i,7] * PE[i,j]
                                        + beta[i,8] * PSM[i,j]
                                        + beta[i,9] * PR[i,j]
                                        + beta[i,10] * Other[i,j]
                                        + beta [i,11] * Baseline_un [i]
                                        + beta[i,12] * CM[i,j] * Baseline_un [i]
                                        + beta[i,13] * TC[i,j] * Baseline_un [i]
                                        + beta[i,14] * EPR[i,j] * Baseline_un [i]
                                        + beta[i,15] * CE[i,j] * Baseline_un [i]
                                        + beta[i,16] * FR[i,j] * Baseline_un [i]
                                        + beta[i,17] * PE[i,j] * Baseline_un [i]
                                        + beta[i,18] * PSM[i,j] * Baseline_un [i]
                                        + beta[i,19] * PR[i,j] * Baseline_un [i]
                                        + beta[i,20] * Other[i,j] * Baseline_un [i]

                        prec.resp[i,j] <- (1/se[i,j])*(1/se[i,j])
                        }
                for(m in 1:20) {
                beta[i,m] ~ dnorm(mu_bar[m], prec[m])
                }
        }
for(i in 60:68) {
                for(j in 1:n_arms[i]) {
                        y[i,j] ~ dnorm(mu[i,j] , prec.resp[i,j])
                        mu[i,j] = beta[i,1] + beta[i,2] * CM[i,j]
                                        + beta[i,3] * TC[i,j]
                                        + beta[i,4] * EPR[i,j]
                                        + beta[i,5] * CE[i,j]
                                        + beta[i,6] * FR[i,j]
                                        + beta[i,7] * PE[i,j]
                                        + beta[i,8] * PSM[i,j]
                                        + beta[i,9] * PR[i,j]
                                        + beta[i,10] * Other[i,j]
                                        + beta [i,11] * Baseline_un [i]
                                        + beta[i,12] * CM[i,j] * Baseline_un [i]
                                        + beta[i,13] * TC[i,j] * Baseline_un [i]
                                        + beta[i,14] * EPR[i,j] * Baseline_un [i]
                                        + beta[i,15] * CE[i,j] * Baseline_un [i]
                                        + beta[i,16] * FR[i,j] * Baseline_un [i]
                                        + beta[i,17] * PE[i,j] * Baseline_un [i]
                                        + beta[i,18] * PSM[i,j] * Baseline_un [i]
                                        + beta[i,19] * PR[i,j] * Baseline_un [i]
                                        + beta[i,20] * Other[i,j] * Baseline_un [i]
                        prec.resp[i,j] <- (1/(se[i,j]*sqrt(corrf[i,j])))*(1/(se[i,j]*sqrt(corrf[i,j])))
                        corrf[i,j] <- 1 + (avg_cluster_size[i,j]-1) * ICC[i,j]
                        ICC[i,j] <- exp(logit_ICC[i,j]) / ( 1 + exp(logit_ICC[i,j]))
                        logit_ICC[i,j] ~ dnorm(-4.16224, (1/(1.8812*1.8812)))
                        }
                for(m in 1:20) {
```

```
			beta[i,m] ~ dnorm(mu_bar[m], prec[m])
			}
		}
	for(i in 69:100) {
			for(j in 1:n_arms[i]) {
				y[i,j] ~ dnorm(mu[i,j] , prec.resp[i,j])
				mu[i,j] = beta[i,1] + beta[i,2] * CM[i,j]
							+ beta[i,3] * TC[i,j]
							+ beta[i,4] * EPR[i,j]
							+ beta[i,5] * CE[i,j]
							+ beta[i,6] * FR[i,j]
							+ beta[i,7] * PE[i,j]
							+ beta[i,8] * PSM[i,j]
							+ beta[i,9] * PR[i,j]
							+ beta[i,10] * Other[i,j]
							+ beta [i,11] * Baseline_un [i]
							+ beta[i,12] * CM[i,j] * Baseline_un [i]
							+ beta[i,13] * TC[i,j] * Baseline_un [i]
							+ beta[i,14] * EPR[i,j] * Baseline_un [i]
							+ beta[i,15] * CE[i,j] * Baseline_un [i]
							+ beta[i,16] * FR[i,j] * Baseline_un [i]
							+ beta[i,17] * PE[i,j] * Baseline_un [i]
							+ beta[i,18] * PSM[i,j] * Baseline_un [i]
							+ beta[i,19] * PR[i,j] * Baseline_un [i]
							+ beta[i,20] * Other[i,j] * Baseline_un [i]
				prec.resp[i,j] <- (1/se[i,j])*(1/se[i,j])
				se[i,j] ~ dunif(0,2)
				}
			for(m in 1:20) {
			beta[i,m] ~ dnorm(mu_bar[m], prec[m])
			}
		}
	for(i in 101:101) {
			for(j in 1:n_arms[i]) {
				y[i,j] ~ dnorm(mu[i,j] , prec.resp[i,j])
				mu[i,j] = beta[i,1] + beta[i,2] * CM[i,j]
							+ beta[i,3] * TC[i,j]
							+ beta[i,4] * EPR[i,j]
							+ beta[i,5] * CE[i,j]
							+ beta[i,6] * FR[i,j]
							+ beta[i,7] * PE[i,j]
							+ beta[i,8] * PSM[i,j]
							+ beta[i,9] * PR[i,j]
							+ beta[i,10] * Other[i,j]
							+ beta [i,11] * Baseline_un [i]
							+ beta[i,12] * CM[i,j] * Baseline_un [i]
							+ beta[i,13] * TC[i,j] * Baseline_un [i]
							+ beta[i,14] * EPR[i,j] * Baseline_un [i]
							+ beta[i,15] * CE[i,j] * Baseline_un [i]
							+ beta[i,16] * FR[i,j] * Baseline_un [i]
							+ beta[i,17] * PE[i,j] * Baseline_un [i]
							+ beta[i,18] * PSM[i,j] * Baseline_un [i]
							+ beta[i,19] * PR[i,j] * Baseline_un [i]
							+ beta[i,20] * Other[i,j] * Baseline_un [i]
				se[i,j] ~ dunif(0, 2)
				prec.resp[i,j] <- (1/(se[i,j]*sqrt(corrf[i,j])))*(1/(se[i,j]*sqrt(corrf[i,j])))
				corrf[i,j] <- 1 + (avg_cluster_size[i,j]-1) * ICC[i,j]
				}
			for(m in 1:20) {
			beta[i,m] ~ dnorm(mu_bar[m], prec[m])
			}
		}
```

```
for(i in 102:114) {
        for(j in 1:n_arms[i]) {
                y[i,j] ~ dnorm(mu[i,j] , prec.resp[i,j])
                mu[i,j] = beta[i,1] + beta[i,2] * CM[i,j]
                                                + beta[i,3] * TC[i,j]
                                                + beta[i,4] * EPR[i,j]
                                                + beta[i,5] * CE[i,j]
                                                + beta[i,6] * FR[i,j]
                                                + beta[i,7] * PE[i,j]
                                                + beta[i,8] * PSM[i,j]
                                                + beta[i,9] * PR[i,j]
                                                + beta[i,10] * Other[i,j]
                                                + beta [i,11] * Baseline_un [i]
                                                + beta[i,12] * CM[i,j] * Baseline_un [i]
                                                + beta[i,13] * TC[i,j] * Baseline_un [i]
                                                + beta[i,14] * EPR[i,j] * Baseline_un [i]
                                                + beta[i,15] * CE[i,j] * Baseline_un [i]
                                                + beta[i,16] * FR[i,j] * Baseline_un [i]
                                                + beta[i,17] * PE[i,j] * Baseline_un [i]
                                                + beta[i,18] * PSM[i,j] * Baseline_un [i]
                                                + beta[i,19] * PR[i,j] * Baseline_un [i]
                                                + beta[i,20] * Other[i,j] * Baseline_un [i]
                se[i,j] ~ dunif(0,2)
                prec.resp[i,j] <- (1/(se[i,j]*sqrt(corrf[i,j])))*(1/(se[i,j]*sqrt(corrf[i,j])))
                corrf[i,j] <- 1 + (avg_cluster_size[i,j]-1) * ICC[i,j]
                ICC[i,j] <- exp(logit_ICC[i,j]) / ( 1 + exp(logit_ICC[i,j]))
                logit_ICC[i,j] ~ dnorm(-4.16224, (1/(1.8812*1.8812)))
                }
        for(m in 1:20) {
        beta[i,m] ~ dnorm(mu_bar[m], prec[m])
        }
    }

        mu_bar[1] ~ dnorm(8,0.01)
        for(m in 2:20) {    mu_bar[m] ~ dnorm(0,0.25) }
        for(m in 1:20) {
                    prec[m] = (1/tau[m]) * (1/tau[m])
                    }
        tau[1] ~ dunif(0,2)
        for(m in 2:20) {    tau[m] ~ dunif(0,2) }
for(m in 1:20) {
    mu_bar_new[m] ~ dnorm(mu_bar[m] , prec[m])
}
}
```

Web Appendix 8. Meta-Regression Model Extension: Effect Modification – Continuous HbA1c

```
model {
	for(i in 1:59) {
		for(j in 1:n_arms[i]) {
			y[i,j] ~ dnorm(mu[i,j] , prec.resp[i,j])
			mu[i,j] = beta[i,1] + beta[i,2] * CM[i,j]
						+ beta[i,3] * TC[i,j]
						+ beta[i,4] * EPR[i,j]
						+ beta[i,5] * CE[i,j]
						+ beta[i,6] * FR[i,j]
						+ beta[i,7] * PE[i,j]
						+ beta[i,8] * PSM[i,j]
						+ beta[i,9] * PR[i,j]
						+ beta[i,10] * Other[i,j]
						+ beta [i,11] * (Baseline_risk_study[i] -
Baseline_risk_sample[i])
						+ beta[i,12] * CM[i,j] * (Baseline_risk_study[i] -
Baseline_risk_sample[i])
						+ beta[i,13] * TC[i,j] * (Baseline_risk_study[i] -
Baseline_risk_sample[i])
						+ beta[i,14] * EPR[i,j] * (Baseline_risk_study[i] -
Baseline_risk_sample[i])
						+ beta[i,15] * CE[i,j] * (Baseline_risk_study[i] -
Baseline_risk_sample[i])
						+ beta[i,16] * FR[i,j] * (Baseline_risk_study[i] -
Baseline_risk_sample[i])
						+ beta[i,17] * PE[i,j] * (Baseline_risk_study[i] -
Baseline_risk_sample[i])
						+ beta[i,18] * PSM[i,j] * (Baseline_risk_study[i] -
Baseline_risk_sample[i])
						+ beta[i,19] * PR[i,j] * (Baseline_risk_study[i] -
Baseline_risk_sample[i])
						+ beta[i,20] * Other[i,j] * (Baseline_risk_study[i] -
Baseline_risk_sample[i])

			prec.resp[i,j] <- (1/se[i,j])*(1/se[i,j])
			}
		for(m in 1:20) {
		beta[i,m] ~ dnorm(mu_bar[m], prec[m])
		}
	}
for(i in 60:68) {
		for(j in 1:n_arms[i]) {
			y[i,j] ~ dnorm(mu[i,j] , prec.resp[i,j])
			mu[i,j] = beta[i,1] + beta[i,2] * CM[i,j]
						+ beta[i,3] * TC[i,j]
						+ beta[i,4] * EPR[i,j]
						+ beta[i,5] * CE[i,j]
						+ beta[i,6] * FR[i,j]
						+ beta[i,7] * PE[i,j]
						+ beta[i,8] * PSM[i,j]
						+ beta[i,9] * PR[i,j]
						+ beta[i,10] * Other[i,j]
						+ beta [i,11] * (Baseline_risk_study[i] -
Baseline_risk_sample[i])
						+ beta[i,12] * CM[i,j] * (Baseline_risk_study[i] -
Baseline_risk_sample[i])
```

```
                                                + beta[i,13] * TC[i,j] * (Baseline_risk_study[i] -
Baseline_risk_sample[i])
                                                + beta[i,14] * EPR[i,j] * (Baseline_risk_study[i] -
Baseline_risk_sample[i])
                                                + beta[i,15] * CE[i,j] * (Baseline_risk_study[i] -
Baseline_risk_sample[i])
                                                + beta[i,16] * FR[i,j] * (Baseline_risk_study[i] -
Baseline_risk_sample[i])
                                                + beta[i,17] * PE[i,j] * (Baseline_risk_study[i] -
Baseline_risk_sample[i])
                                                + beta[i,18] * PSM[i,j] * (Baseline_risk_study[i] -
Baseline_risk_sample[i])
                                                + beta[i,19] * PR[i,j] * (Baseline_risk_study[i] -
Baseline_risk_sample[i])
                                                + beta[i,20] * Other[i,j] * (Baseline_risk_study[i] -
Baseline_risk_sample[i])

                        prec.resp[i,j] <- (1/(se[i,j]*sqrt(corrf[i,j])))*(1/(se[i,j]*sqrt(corrf[i,j])))
                        corrf[i,j] <- 1 + (avg_cluster_size[i,j]-1) * ICC[i,j]
                        ICC[i,j] <- exp(logit_ICC[i,j]) / ( 1 + exp(logit_ICC[i,j]))
                        logit_ICC[i,j] ~ dnorm(-4.16224, (1/(1.8812*1.8812)))
                        }
            for(m in 1:20) {
            beta[i,m] ~ dnorm(mu_bar[m], prec[m])
            }
      }
for(i in 69:100) {
            for(j in 1:n_arms[i]) {
                        y[i,j] ~ dnorm(mu[i,j] , prec.resp[i,j])
                        mu[i,j] = beta[i,1] + beta[i,2] * CM[i,j]
                                                + beta[i,3] * TC[i,j]
                                                + beta[i,4] * EPR[i,j]
                                                + beta[i,5] * CE[i,j]
                                                + beta[i,6] * FR[i,j]
                                                + beta[i,7] * PE[i,j]
                                                + beta[i,8] * PSM[i,j]
                                                + beta[i,9] * PR[i,j]
                                                + beta[i,10] * Other[i,j]
                                                + beta [i,11] * (Baseline_risk_study[i] -
Baseline_risk_sample[i])
                                                + beta[i,12] * CM[i,j] * (Baseline_risk_study[i] -
Baseline_risk_sample[i])
                                                + beta[i,13] * TC[i,j] * (Baseline_risk_study[i] -
Baseline_risk_sample[i])
                                                + beta[i,14] * EPR[i,j] * (Baseline_risk_study[i] -
Baseline_risk_sample[i])
                                                + beta[i,15] * CE[i,j] * (Baseline_risk_study[i] -
Baseline_risk_sample[i])
                                                + beta[i,16] * FR[i,j] * (Baseline_risk_study[i] -
Baseline_risk_sample[i])
                                                + beta[i,17] * PE[i,j] * (Baseline_risk_study[i] -
Baseline_risk_sample[i])
                                                + beta[i,18] * PSM[i,j] * (Baseline_risk_study[i] -
Baseline_risk_sample[i])
                                                + beta[i,19] * PR[i,j] * (Baseline_risk_study[i] -
Baseline_risk_sample[i])
                                                + beta[i,20] * Other[i,j] * (Baseline_risk_study[i] -
Baseline_risk_sample[i])

                        prec.resp[i,j] <- (1/se[i,j])*(1/se[i,j])
                        se[i,j] ~ dunif(0,2)
                        }
```

```
            for(m in 1:20) {
            beta[i,m] ~ dnorm(mu_bar[m], prec[m])
            }
      }
for(i in 101:101) {
            for(j in 1:n_arms[i]) {
                  y[i,j] ~ dnorm(mu[i,j] , prec.resp[i,j])
                  mu[i,j] = beta[i,1] + beta[i,2] * CM[i,j]
                                          + beta[i,3] * TC[i,j]
                                          + beta[i,4] * EPR[i,j]
                                          + beta[i,5] * CE[i,j]
                                          + beta[i,6] * FR[i,j]
                                          + beta[i,7] * PE[i,j]
                                          + beta[i,8] * PSM[i,j]
                                          + beta[i,9] * PR[i,j]
                                          + beta[i,10] * Other[i,j]
                                          + beta [i,11] * (Baseline_risk_study[i] -
Baseline_risk_sample[i])
                                          + beta[i,12] * CM[i,j] * (Baseline_risk_study[i] -
Baseline_risk_sample[i])
                                          + beta[i,13] * TC[i,j] * (Baseline_risk_study[i] -
Baseline_risk_sample[i])
                                          + beta[i,14] * EPR[i,j] * (Baseline_risk_study[i] -
Baseline_risk_sample[i])
                                          + beta[i,15] * CE[i,j] * (Baseline_risk_study[i] -
Baseline_risk_sample[i])
                                          + beta[i,16] * FR[i,j] * (Baseline_risk_study[i] -
Baseline_risk_sample[i])
                                          + beta[i,17] * PE[i,j] * (Baseline_risk_study[i] -
Baseline_risk_sample[i])
                                          + beta[i,18] * PSM[i,j] * (Baseline_risk_study[i] -
Baseline_risk_sample[i])
                                          + beta[i,19] * PR[i,j] * (Baseline_risk_study[i] -
Baseline_risk_sample[i])
                                          + beta[i,20] * Other[i,j] * (Baseline_risk_study[i] -
Baseline_risk_sample[i])

                  se[i,j] ~ dunif(0, 2)
                  prec.resp[i,j] <- (1/(se[i,j]*sqrt(corrf[i,j])))*(1/(se[i,j]*sqrt(corrf[i,j])))
                  corrf[i,j] <- 1 + (avg_cluster_size[i,j]-1) * ICC[i,j]
                  }
            for(m in 1:20) {
            beta[i,m] ~ dnorm(mu_bar[m], prec[m])
            }
      }
for(i in 102:114) {
            for(j in 1:n_arms[i]) {
                  y[i,j] ~ dnorm(mu[i,j] , prec.resp[i,j])
                  mu[i,j] = beta[i,1] + beta[i,2] * CM[i,j]
                                          + beta[i,3] * TC[i,j]
                                          + beta[i,4] * EPR[i,j]
                                          + beta[i,5] * CE[i,j]
                                          + beta[i,6] * FR[i,j]
                                          + beta[i,7] * PE[i,j]
                                          + beta[i,8] * PSM[i,j]
                                          + beta[i,9] * PR[i,j]
                                          + beta[i,10] * Other[i,j]
                                          + beta [i,11] * (Baseline_risk_study[i] -
Baseline_risk_sample[i])
                                          + beta[i,12] * CM[i,j] * (Baseline_risk_study[i] -
Baseline_risk_sample[i])
```

```
                                                                + beta[i,13] * TC[i,j] * (Baseline_risk_study[i] -
Baseline_risk_sample[i])
                                                                + beta[i,14] * EPR[i,j] * (Baseline_risk_study[i] -
Baseline_risk_sample[i])
                                                                + beta[i,15] * CE[i,j] * (Baseline_risk_study[i] -
Baseline_risk_sample[i])
                                                                + beta[i,16] * FR[i,j] * (Baseline_risk_study[i] -
Baseline_risk_sample[i])
                                                                + beta[i,17] * PE[i,j] * (Baseline_risk_study[i] -
Baseline_risk_sample[i])
                                                                + beta[i,18] * PSM[i,j] * (Baseline_risk_study[i] -
Baseline_risk_sample[i])
                                                                + beta[i,19] * PR[i,j] * (Baseline_risk_study[i] -
Baseline_risk_sample[i])
                                                                + beta[i,20] * Other[i,j] * (Baseline_risk_study[i] -
Baseline_risk_sample[i])
                        se[i,j] ~ dunif(0,2)
                        prec.resp[i,j] <- (1/(se[i,j]*sqrt(corrf[i,j])))*(1/(se[i,j]*sqrt(corrf[i,j])))
                        corrf[i,j] <- 1 + (avg_cluster_size[i,j]-1) * ICC[i,j]
                        ICC[i,j] <- exp(logit_ICC[i,j]) / ( 1 + exp(logit_ICC[i,j]))
                        logit_ICC[i,j] ~ dnorm(-4.16224, (1/(1.8812*1.8812)))
                        }
                for(m in 1:20) {
                beta[i,m] ~ dnorm(mu_bar[m], prec[m])
                }
        }
                mu_bar[1] ~ dnorm(8,0.01)
                for(m in 2:20) {        mu_bar[m] ~ dnorm(0,0.25) }
                for(m in 1:20) {
                                        prec[m] = (1/tau[m]) * (1/tau[m])
                                        }
                tau[1] ~ dunif(0,2)
                for(m in 2:20) {        tau[m] ~ dunif(0,2) }


                rk <-rank(mu_bar[2:20])
for(m in 1:20) {
        mu_bar_new[m] ~ dnorm(mu_bar[m] , prec[m])
}
}
```

Web Table 3. Summary of Results Comparing Meta-Regression of Study-Level Differences vs. Response Surface Meta-regression of Arm Means

QI strategy	Meta-regression of differences (study-level)		Meta-regression of means (arm-level)	
	**Median**	**95% CrI**	**Median**	**95% CrI**
CM	-0.12	-0.94, 0.67	0.03	-0.12, 0.17
TC	-0.32	-1.09, 0.44	-0.36	-0.54, -0.18
EPR	-0.14	-1.31, 1.02	-0.15	-0.38, 0.07
CE	-0.17	-1.37, 1.03	-0.17	-0.44, 0.08
FR	-0.18	-1.10, 0.73	-0.24	-0.43, -0.06
PE	-0.15	-1.04,0.78	-0.10	-0.28, 0.08
PSM	-0.16	-1.08, 0.77	-0.17	-0.37, 0.01
PR	0.09	-1.04, 1.20	-0.00	-0.23, 0.22
Other[a]	0.03	-0.98, 1.07	0.02	-0.20, 0.18

Abbreviations: CM=case management, CE=clinician education, EPR=electronic patient registry, FR=facilitated relay, TC=team change, PE=patient education, PR=patient reminders, PSM=promotion of self-management, QI=quality improvement.

[a] Other: Combined category for infrequently evaluated component including audit and feedback, clinician reminders, financial incentives and continuous quality improvement.

We used the following prior distributions in the Bayesian analyses: $\beta_0 \sim N(8,100)$; $\tau_0 \sim U(0,2)$; $\beta_k \sim N(0,4)$; $\tau_{\beta_k} \sim U(0,2)$.

References for Web Appendix

1. Freeman SC, Scott NW, Powell R, et al. Component network meta-analysis identifies the most effective components of psychological preparation for adults undergoing surgery under general anesthesia. *J Clin Epidemiol* 2018;98:105-16.
2. Welton NJ, Caldwell DM, Adamopoulos E, et al. Mixed treatment comparison meta-analysis of complex interventions: psychological interventions in coronary heart disease. *Am J Epidemiol* 2009;169(9):1158-65.
3. Gelman A, Stevens M, Chan V. Regression Modeling and Meta-Analysis for Decision Making. *Journal of Business & Economic Statistics* 2003;21(2):213-25.
4. Caldwell DM, Welton NJ. Approaches for synthesising complex mental health interventions in meta-analysis. *Evid Based Ment Health* 2016;19(1):16-21.
5. Welton NJ, Sutton AJ, Cooper NJ, et al. *Evidence synthesis for decision making in healthcare*. Chichester: Wiley; 2012.
6. *Cochrane Handbook for Systematic Reviews of Interventions Version 5.1.0 [updated March 2011]*. The Cochrane Collaboration; 2011.
7. Turner RM, Thompson SG, Spiegelhalter DJ. Prior distributions for the intracluster correlation coefficient, based on multiple previous estimates, and their application in cluster randomized trials. *Clin Trials* 2005;2(2):108-18.
8. Danko KJ. *Methods for Optimizing Evidence Syntheses of Complex Interventions: Case Study of a Systematic Review and Meta-Analysis of Diabetes Quality Improvement Trials*. Ottawa: University of Ottawa; 2018.
9. Hozo SP, Djulbegovic B, Hozo I. Estimating the mean and variance from the median, range, and the size of a sample. *BMC Med Res Methodol* 2005;5:13.
10. Chapter 7: Selecting studies and collecting data In: Higgins J GS, ed. *Cochrane Handbook for Systematic Reviews of Interventions Version 510 [updated March 2011]*. Chichester: The Cochrane Collaboration, 2011.
